# Prophage dynamics in gastric and enterohepatic environments: unraveling ecological barriers and adaptive transitions

**DOI:** 10.1093/ismeco/ycaf017

**Published:** 2025-02-04

**Authors:** Marta Proença, Luís Tanoeiro, James G Fox, Filipa F Vale

**Affiliations:** Pathogen Genomics and Translational Microbiology Lab, BioISI – Instituto de Biosistemas e Ciências Integrativas, Faculdade de Ciências, Universidade de Lisboa, 1749-016 Lisboa, Portugal; Research Institute for Medicines (iMed-ULisboa), Faculty of Pharmacy, Universidade de Lisboa, 1649-003 Lisboa, Portugal; Pathogen Genomics and Translational Microbiology Lab, BioISI – Instituto de Biosistemas e Ciências Integrativas, Faculdade de Ciências, Universidade de Lisboa, 1749-016 Lisboa, Portugal; Research Institute for Medicines (iMed-ULisboa), Faculty of Pharmacy, Universidade de Lisboa, 1649-003 Lisboa, Portugal; Division of Comparative Medicine, Massachusetts Institute of Technology, Cambridge, MA 02139-4307, United States; Pathogen Genomics and Translational Microbiology Lab, BioISI – Instituto de Biosistemas e Ciências Integrativas, Faculdade de Ciências, Universidade de Lisboa, 1749-016 Lisboa, Portugal; Research Institute for Medicines (iMed-ULisboa), Faculty of Pharmacy, Universidade de Lisboa, 1649-003 Lisboa, Portugal

**Keywords:** *Helicobacter*bacteriophage, phage–host interactions, prophage diversity, gastric and enterohepatic species

## Abstract

Phage predation plays a critical role in shaping bacterial genetic diversity, with prophages playing a comparable role. However, the prevalence and genetic variability of prophages within the *Helicobacter* genus remain inadequately studied. *Helicobacter* species are clinically significant and occupy distinct digestive system regions, with gastric species (e.g. *Helicobacter pylori*) residing in the gastric mucosa and enterohepatic species colonizing the liver and intestines of various vertebrates. Here, we address this knowledge gap by analyzing prophage presence and diversity across 343 non-*pylori Helicobacter* genomes, mapping their distribution, comparing genomic features between gastric and enterohepatic prophages, and exploring their evolutionary relationships with hosts. We identified and analyzed a catalog of 119 new complete and 78 incomplete prophages. Our analysis reveals significant differences between gastric and enterohepatic species. Gastric prophages exhibit high synteny, and cluster in a few groups, indicating a more conserved genetic structure. In contrast, enterohepatic prophages show greater diversity in gene order and content, reflecting their adaptation to varied host environments. *Helicobacter cinaedi* stands out, harboring a large number of prophages among the enterohepatic species, forming a distinct cohesive group. Phylogenetic analyses reveal a co-evolutionary relationship between several prophages and their bacterial hosts—though exceptions, such as the enterohepatic prophages from *H. canis*, *H. equorum*, *H. jaachi*, and the gastric prophage from *H. himalayensis*—suggesting more complex co-evolutionary dynamics like host jumps, recombination, and horizontal gene transfer. The insights gained from this study enhance our understanding of prophage dynamics in *Helicobacter*, emphasizing their role in bacterial adaptation, virulence, and host specificity.

## Introduction

The *Helicobacter* genus, part of the *Helicobacteraceae* family in the *Epsilonproteobacteria* class, includes microaerophilic species that are often catalase-, oxidase-, and urease-positive [1]. Currently, 54 *Helicobacter* species are officially recognized by the List of Prokaryotic names with Standing in Nomenclature. These bacteria inhabit the gastric and enterohepatic tracts of various vertebrates, including mammals, birds, and reptiles. They are adapted to certain mammalian hosts, with gastric species unable to infect the liver or intestines, and vice versa [2, 3]. Notably, *Helicobacter pylori* is recognized for its association with human gastric diseases such as gastritis, peptic ulcers, and gastric cancer. The interactions between *H. pylori* and its human host highlight the complex co-evolutionary dynamics shaped by human migrations and population movements, reflecting how this bacterium has adapted alongside humans over time [4–6]. This co-evolution is driven by various ecological and evolutionary pressures, influencing both bacterial diversity and host adaptation mechanisms [7, 8].

Prophages, the integrated forms of bacteriophages within bacterial genomes, are crucial players in the evolutionary and ecological landscape of bacteria. These genetic elements contribute to horizontal gene transfer, genetic diversification, and the adaptive capabilities of their bacterial hosts. They influence bacterial pathogenicity, resistance mechanisms, and ecological adaptation by providing new genetic material and facilitating gene exchange between bacterial populations [9–11] Accordingly, cargo genes were shown to be present in the *H. pylori* prophages [12]. In *H. pylori*, prophages are not just passive genomic elements but active participants in the evolutionary narrative [12, 13]. For instance, the presence of prophage genes in *H. pylori* correlates with the bacterium’s phylogeographic distribution, suggesting a long-term co-evolutionary relationship driven by ecological and evolutionary pressures [14–16]. *Helicobacter pylori* prophages are mainly incomplete, and the inactivation mechanisms seem to involve genome rearrangement, fusion with other mobile elements, and pseudogene accumulation [12].

Despite extensive studies on *H. pylori*, the co-evolutionary dynamics between non-*pylori Helicobacter* species and their prophages remain largely unexplored. While some prophages have been identified in species like *H. acinonychis* [17], *H. bizzozeronii* [18], *H. felis* [19], and *H. pullorum* [20], their characterization has not been the primary focus of these studies and they have not been thoroughly examined. The prophages in non-*pylori Helicobacter* species may play pivotal roles in host adaptation and specificity, potentially shaping ecological interactions and evolutionary trajectories. However, a broader, in-depth analysis of prophages across diverse *Helicobacter* species is essential to fully unravel the extent of these dynamics. This study addresses a critical knowledge gap by investigating prophages across a broad spectrum of non-*pylori Helicobacter* species. Through the analysis of 343 bacterial genomes from the genus *Helicobacter*, it aims to identify and characterize prophage elements, shedding light on their role in the co-evolutionary dynamics between prophages and their bacterial hosts. Key objectives include mapping the distribution of prophages within the genus, comparing genomic features between prophages from gastric and enterohepatic species, and uncovering the evolutionary relationships between prophages and their hosts. This work offers valuable insights into the ecological and evolutionary significance of prophage–bacteria co-evolution in *Helicobacter*, advancing our understanding of bacterial adaptation, virulence, and ecology.

## Materials and methods

### Collection of non-*pylori Helicobacter* genomes

To compile all available non-*pylori Helicobacter* genomes (Table S1), the existing list from Mannion *et al.* 2018 [21] and all available genomes in the Bacterial and Viral Bioinformatics Resource Center (BV-BRC) (https://www.bv-brc.org/) were cross-referenced to obtain a comprehensive list of publicly available genomes as of 28 September 2021. Genomes without annotation were submitted for annotation in RAST v2.0 (https://rast.nmpdr.org/) [22]. Additionally, we have included five *H. pylori* genomes that carry prophages: *H. pylori* De-M53-M (accession number JAHGYT010000000), *H. pylori* Fr-B58-M (accession number JAHGYS010000000), *H. pylori* B45 harboring prophage phiHP33 (accession number AFAO01000000), *H. pylori* Pt-1918-U (accession number JAHGYN010000000), and *H. pylori* Pt-1293-U (accession number JAHGYP010000000).

### Prophage identification

To identify possible prophages in the selected genomes of non-*pylori Helicobacter* species, three different approaches were employed. Two of these approaches utilized specific prophage detection software: PHASTER [23] and Prophage Hunter [24]. The third approach was a snowball method that used BLAST searches with manually curated *Helicobacter* prophages as queries, considering at least 50% of query coverage. Using Geneious Prime 2022.0.1 (https://www.geneious.com/), a manual curation of the identified prophages was performed to ensure more accurate identification and delimitation of each prophage sequence. This process, as previously described by Vale *et al.* [12, 15], involved validating the sequences by identifying typical phage genes (such as integrase, primase, helicase, portal protein, terminase, lysin, holin, and structural proteins) and ensuring the regions mostly lacked bacterial genes. After manual curation prophages were categorized as complete or incomplete. Complete prophages were those with a considerable genome size (>20 kb), clearly delimited by bacterial genes, and those with genome sizes >30 kb that were not fully complete due to contig breaks. Incomplete prophages were delimited but had a genome size <20 kb or were partial prophage genome fragments that could not be scaffolded. It is important to note that regions did not need to exhibit all the specified features to be considered a prophage. Given that the identified prophage sequences contained a high number of hypothetical proteins, at least one phage gene should be present to pass manual curation. Finally, the snowball approach involved using each complete prophage identified up to that point as a query template to search for additional prophages within the non-*pylori Helicobacter* genome set using BLASTn [25]. The newly identified prophages from BLASTn were then manually curated as described above. For comparative genomics purposes five *H. pylori* prophages were included: DeM53M (accession number KX119205.1), FrB58M (accession number KX119193.1), phiHP33 (accession number NC_016568.1), Pt1918U (accession number KX119192.1), and Pt1293U (accession number KX119202.1).

### Prophage characterization

Statistical measures included mean lengths, % GC content, and the number of complete prophages, analyzed by species and grouped as gastric or enterohepatic. Prophages were classified using Virfam [26] based on tail characteristics and head-to-tail connection proteins, categorizing them into *Siphoviridae*, *Myoviridae*, and *Podoviridae* families. Although morphology-based classifications were abolished in 2022 [27], this classification helps in comparing with previous studies. The prophages were analyzed using the taxMyPhage tool to attempt classification into genera and species based on genomic similarity [28].

Prophage-shared orthologous genes were identified by Proteinortho v5.16 [29] using default parameters: 25% identity, 50% coverage, and a BLAST E-value threshold of 1e-05. Shared orthologous genes improved annotation was used to identify genes coding hypothetical proteins, using BLASTp [25] searches against the National Center for Biotechnology Information (NCBI) database and Phyre2 [30] for structural and functional insights. Shared orthologous genes were annotated using Pharokka v.1.3.2 [31] with Prodigal v.2.6.3 [32], on the Galaxy platform [33]. Defense and anti-defense genes were analyzed via DefenseFinder webservice [34, 35]. Shannon diversity and equitability indices were calculated for shared orthologous genes in gastric and enterohepatic prophages.

### Phylogenetic analysis of prophages and hosts

Phylogenetic trees were constructed separately for all complete prophages, for complete gastric prophages, and for complete enterohepatic prophages. Additionally, separate phylogenetic trees were generated for the host bacteria carrying these prophages, with one tree for all host bacteria, one for those harboring gastric prophages, and one for those with enterohepatic prophages. Briefly, FASTA nucleotide files for the different groups were aligned using MAFFT v7 [36]. Alignments for complete prophages were performed on entire genomes, as they lacked shared orthologous genes, while alignments for host bacteria focused on shared orthologous genes identified by Proteinortho v5.16 [29]. Phylogenetic trees were generated with FastTree v2 [37], utilizing the maximum-likelihood algorithm for constructing large phylogenies and estimating confidence. The resulting trees were customized using ITOL v5 [38].

### Network analysis of shared genes

Proteinortho v5.16 [29] was employed to identify the orthologous genes shared across the prophages, providing a basis for understanding the genetic relationships and functional similarities. To explore the gene composition and functional relationships of prophages, network analysis was conducted using Cytoscape 3.5 [39]. This analysis focused on identifying and visualizing shared orthologous genes among complete prophages. Networks were constructed based on the number of shared orthologous genes, with separate networks created for those sharing at least 5, 10, or 20 orthologous genes.

### Congruence between phylogenetic trees

Congruence between phylogenetic trees measures how well nodes from one tree align with corresponding nodes in another, indicating potential co-speciation. To assess cospeciation and global host–prophage co-evolution, the Procrustes approach to cophylogeny (PACo) was used [40]. This method applies Procrustes analysis to compare distance matrices derived from the phylogenetic trees of hosts and prophages, using binary matrices to denote prophage presence in hosts. PACo calculates patristic distance, the smallest cumulative branch length needed to get from one tip/node to the other, to evaluate whether prophage phylogeny significantly correlates with host phylogeny. It uses a statistic m^2^_XY_ to determine if the prophage’s phylogeny depends significantly in the host’s, and a *P*-value is calculated.

Additionally, the ParaFit test [41] was employed to further explore host–prophage co-evolution. It uses the same phylogenetic trees and binary matrices to convert them into matrices representing phylogenies then estimates global and individual association statistics using permutation tests with 999 permutations. ParaFit provides both global results (ParaFitGlobal) and individual link assessments, with *P*-values indicating statistical significance.

### Prophage synteny analysis

Synteny analysis assesses the conservation of homologous gene blocks and gene order across prophage genomes. To perform synteny analysis for gastric, enterohepatic, and all prophages, Clinker and clustermap.js were utilized [42]. Clinker processes GenBank files, aligning amino acid sequences with the BioPython package and defaulting to the BLOSUM62 substitution matrix. Clusters are organized hierarchically using a modified MultiGeneBlast formula, which incorporates syntenic conservation and sequence similarity, and the similarity matrix is created using the Ward variance minimization algorithm.

## Results

### Collection of non-*pylori Helicobacter* genomes

The initial list of non-*pylori Helicobacter* genomes was derived from Mannion *et al.* (2018), which included 110 genomes. A second list was obtained from BV-BRC by searching for “Helicobacter” yielding 2454 genomes. These lists were then merged, excluding *H. pylori* genomes, non-*Helicobacter* genomes, and duplicates. The final curated list comprised 343 genomes non-*pylori Helicobacter* genomes, with the number of strains for each species detailed in Table 1. The species with most genomes are *H. cinaedi* (61 genomes), followed by *H. suis* (34 genomes), *H. pullorum* (28 genomes), *H. felis* (23 genomes), *H. bilis* (13 genomes), and *H. bizzozeronii* (11 genomes). All other species have fewer than 10 genomes. Additionally, 51 genomes are from unidentified species. Within the dataset, there are 31 enterohepatic species and 13 gastric species.

**Table 1:** Number of genomes analyzed per species (enterohepatic: 183 genomes; gastric: 109 genomes; NA: 51 genomes) and number of genomes with prophages and number of prophages, sorted by both enterohepatic and gastric species for complete or incomplete prophages.

			Complete prophages		Incomplete prophages	
	Species	Genome number	Number of genomes with prophages	Number of prophages	Number of genomes with prophages	Number of prophages
**Enterohepatic**	*Candidatus H.* avicola^a^	1	0	0	0	0
	*Candidatus H.* avistercoris^a^	1	0	0	1	1
	*H. anseris*	2	0	0	0	0
	*H. apodemos* ^a^	3	1	1	0	0
	*H. aurati*	2	0	0	0	0
	*H. bilis*	13	0	0	3	3
	*H. brantae*	1	0	0	0	0
	*H. canadensis*	5	0	0	0	0
	*H. canis*	5	5	5	0	0
	*H. cholecystus*	3	0	0	0	0
	*H. cinaedi*	61	57	58	0	0
	*H. equorum*	2	1	1	0	0
	*H. fennelliae*	5	0	0	0	0
	*H. ganmani*	2	1	1	0	0
	*H. hepaticus*	2	0	0	2	2
	*H. jaachi*	1	1	1	0	0
	*H. japonicus*	2	0	0	1	2
	*H. macacae*	2	0	0	0	0
	*H. magdeburgensis* ^a^	2	0	0	1	1
	*H. marmotae*	2	0	0	0	0
	*H. mesocricetorum*	1	0	0	1	1
	*H. muridarum*	5	0	0	5	6
	*H. pametensis*	4	1	1	0	0
	*H. pullorum*	28	7	8	6	6
	*H. rappini* ^a^	1	0	0	0	0
	*H. rodentium*	2	1	1	0	0
	*H. sanguini*	5	0	0	0	0
	*H. trogontum*	5	2	2	0	0
	*H. typhlonius*	6	0	0	0	0
	*H. valdiviensis*	1	0	0	1	1
	*H. winghamensis* ^a^	8	0	0	7	8
**Gastric**	*H. acinonychis*	8	4	4	0	0
	*H. ailurogastricus*	6	3	3	2	2
	*H. baculiformis*	1	0	0	1	1
	*H. bizzozeronii*	11	5	5	1	1
	*H. cetorum*	5	2	2	2	3
	*H. cynogastricus*	1	0	0	0	0
	*H. felis*	23	1	1	21	24
	*H. heilmannii*	9	8	8	0	0
	*H. himalayensis*	1	1	1	0	0
	*H. mehlei*	1	0	0	0	0
	*H. mustelae*	3	0	0	0	0
	*H. salomonis*	6	4	4	1	1
	*H. suis*	34	0	0	1	1
**NA**	*Helicobacter* sp.	50	8	10	13	13
	Uncultured *Helicobacter* sp.	1	1	2	1	1
Total		343	114	119	71	78

NA, not available. When the number of prophages exceeds the number of bacterial genomes of a species, this indicates that some genomes contain more than one prophage.

a Species name not validly published (*source:*  https://www.bacterio.net/).

### Identification of prophages and comparative analysis

The 343 genomes of non-*pylori Helicobacter* species were analyzed using PHASTER [23], which identified 518 prophage regions. Of these, 483 (93%) were incomplete, 29 (6%) were questionable, and 6 (1%) were intact, according to PHASTER terminology. After manual curation, we correctly delimited and kept 58 complete and 77 incomplete prophages. The same genomes were then analyzed with Prophage Hunter [24], which detected 88 active prophage regions, according to Prophage Hunter terminology. Manual curation revealed 10 complete and 1 incomplete prophage, including 9 prophages also identified by PHASTER. This brought the total to 68 complete and 78 incomplete prophages. Using the 68 complete prophages as queries in BLASTn [25], an additional 51 complete prophages were identified, resulting in a total of 119 complete prophages across 18 of the 44 species analyzed and 78 incomplete prophages across 17 of the 44 species analyzed. In total, 29 different species contained either complete or incomplete prophages (Table 1). In summary, of the complete prophages used in further analysis, 49% were identified by PHASTER, 8% by Prophage Hunter, and 43% by BLASTn.

For complete prophages (Table 1) among enterohepatic species, all 5 analyzed genomes of *H. canis* harbored prophages while the majority of prophages were identified in *H. cinaedi* (58 prophages in 61 genomes). Among gastric species, all eight *H. heilmannii* genomes had prophages. Every complete prophage found was named after the genome strain name (Table S2). Statistics on sequence length and GC content for the complete prophages are shown in Figs S1 and S2 and Table 2. Details on morphotype prediction are provided in the supplementary material and Table S3.

**Table 2:** Mean sequence length (bp) and mean GC content (%) of the complete prophages and their genome hosts sorted by species.

			Genome hosts		Complete prophages		
Species	Group	Number of complete Prophages	Mean sequence length (bp)	Mean GC (%)	Mean sequence length (bp)	Mean GC (%)	Length (%) prophage/genome
*H. apodemus*	E	1	2 068 160.00	33.00	40 595.00	33.90	1.96
*H. canis*	E	5	1 965 572.00	44.98	42 409.80	45.44	2.19
*H. cinaedi*	E	58	2 122 817.95	38.48	24 851.45	35.92^**^	1.18
*H. equorum*	E	1	1 666 688.00	37.40	33 365.00	34.70	2.00
*H. felis*	E	1	1 672 681.00	44.50	29 904.00	44.90	1.79
*H. ganmani*	E	1	1 808 803.00	37.00	47 136.00	37.70	2.61
*H. pametensis*	E	1	1 918 356.00	55.10	40 715.00	53.00	2.12
*H. pullorum*	E	8	1 710 123.00	34.77	37 388.75	34.04	2.25
*H. rodentium*	E	1	1 810 652.00	37.00	27 342.00	35.40	2.02
*H. trogontum*	E	2	2 418 761.50	33.15	31 573.50	34.05^*^	1.63
*H. acinonychis*	G	4	1 538 161.00	38.15	28 542.50	42.35^**^	1.86
*H. ailurogastricus*	G	3	1 581 241.00	47.60	30 595.33	48.83^**^	1.93
*H. bizzozeronii*	G	5	1 781 438.00	45.88	36 806.00	46.16^**^	2.06
*H. cetorum*	G	2	1 878 391.00	34.60	36 064.50	32.55^*^	1.92
*H. heilmannii*	G	8	1 631 346.25	47.78	48 015.25	46.41^**^	2.98
*H. himalayensis*	G	1	1 829 936.00	39.90	39 255.00	36.60	1.57
*H. jaachi*	G	1	1 904 650.00	41.00	29 954.00	40.90	1.57
*H. pylori*	G	5	1 613 085.60	38.94	26 802.60	36.52^**^	1.66
*H. salomonis*	G	4	1 629 548.25	45.93	33 578.50	47.40	1.93
*Helicobacter* sp*.*	NA	10	1 845 338.75	37.96	31 569.10	37.56	1.69
*u.Helicobacter* sp*.*	NA	2	2 013 685.00	38.70	34 605.50	37.25	1.62
**Total**		124	1 935 192.59	39.91	30 672.76	38.50	1.64

Percentage of the length prophage/genome is also present. u means uncultured. u, uncultured; G, gastric; E, enterohepatic; NA, not available. For the 119 complete prophages, the mean sequence length is 30.8 kb [standard deviation (SD) 9.0 kb] with a mean GC content of 38.58% (SD 5.0%), while bacterial genomes averaged 1.95 Mb (SD 0.24 Mb) with a mean GC content of 39.79% (SD 4.3%); including five *H. pylori* prophages (phiHP33, Pt1293U, DeM53M, FrB58M, and Pt1918U) the average prophage length was 30.7 kb (SD 8.9 kb) with 38.50% GC content (SD 4.87%), while bacterial genomes averaged 1.93 Mb (SD 0.24 Mb) with 39.76% GC content (SD 4.22%).

^*^Indicates statistical significance at the *P* < .01;

^**^Indicates statistical significance at the *P* < .001, between bacterial and prophage GC content (*t*-test).

### Prophage and bacteria shared orthologous genes

Among the 124 complete prophages, 457 orthologous genes were identified, with no gene common to all. For gastric and enterohepatic prophages, 169 and 275 orthologous genes were found, respectively. Singleton genes were not included in these counts. The most frequent orthologue, possibly coding for the portal protein, appeared in 84 sequences. The second most common, coding for a hypothetical protein, was found in 63 sequences, and the third, coding for a DNA-binding protein, was in 61 sequences. Even among the *H. cinaedi* prophages, no common orthologous gene was identified out of 109 orthologues.

Out of the 457 orthologous genes, functions could be predicted for only 123, as shown in Table S4. Only a complete defense and a complete antidefense system were predicted (full details are available in the supplementary material). In the 119 host bacterial genomes, 181 orthologous genes were common to all strains (1.94%, 181/9345). For the 77 genomes in the enterohepatic group, 264 orthologous genes were identified (4.62%, 264/5720). The 33 genomes in the gastric group had 673 common orthologous genes (15.58%, 673/4321). Singleton genes were not included in these counts. The enterohepatic species prophages are more diverse than those of gastric species concerning the orthologous genes identified. This is reflected in the higher number of common orthologous genes in gastric genomes compared to enterohepatic genomes. For *H. cinaedi* species, with 57 genomes, 1359 common orthologous genes were found out of 3434 total orthologous genes.

Enterohepatic prophages show slightly higher diversity (Shannon diversity index H′ = 4.989) but lower evenness (Shannon equitability index: 0.861) than gastric prophages (H′ = 4.959, equitability 0.931), indicating a more uneven gene distribution.

### Phylogenetic analysis

**Figure 1:**
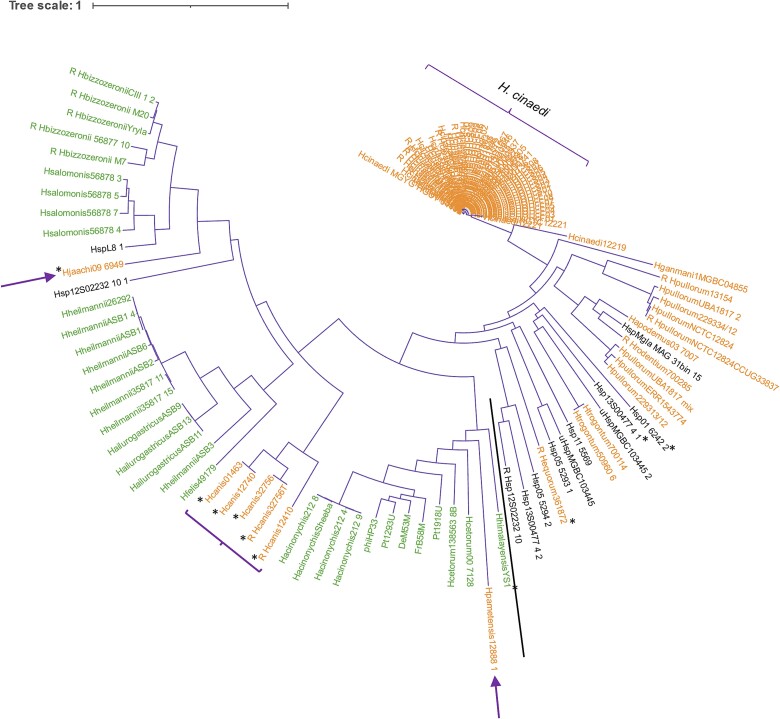
Phylogenetic tree based on prophage complete genomes. Tree obtained using the FastTreeMP v2.1.11 program and visualized with software iTOL v5. Green indicates gastric species; orange, enterohepatic species; black, *Helicobacter* sp. The letter R behind the names represents the prophages where the sequence was reversed in the alignment; the black line does the separation between the gastric and enterohepatic groups and the bold purple arrows and braces represent the enterohepatic prophages in the gastric side. The black ^*^ represent the strains that are not in co-evolution.

The phylogenetic tree of described prophages reveals two main clusters: enterohepatic prophages (orange) and gastric prophages (green) (Fig. 1). Most *H. cinaedi* prophages are closely related within the enterohepatic cluster, except for Hcinaedi12219, HcinaediNCTC12221, and Hcinaedi12221, whose host species in these three cases were isolated from dogs (Table S2). The phylogenetic tree (Fig. 1) shows that HcinaediNCTC12221 and Hcinaedi12221 are very closely related and cluster near the prophages whose host species has been isolated from humans, while Hcinaedi12219 is more distantly related. These findings suggest that interspecies transmission events between humans and dogs have occurred and that these lineages, including their prophages, may be diverging over time. *Helicobacter pylori* prophages are within the gastric cluster. Unexpectedly, three enterohepatic species have prophages (seven in total) that cluster within the gastric group, marked by purple arrows and brackets: *H. jaachi* (Hjaachi09_6949), *H. canis* (Hcanis01463, Hcanis12740, Hcanis32756, Hcanis32756T, Hcanis12410), and *H. pametensis* (Hpametensis12888_1).

**Figure 2:**
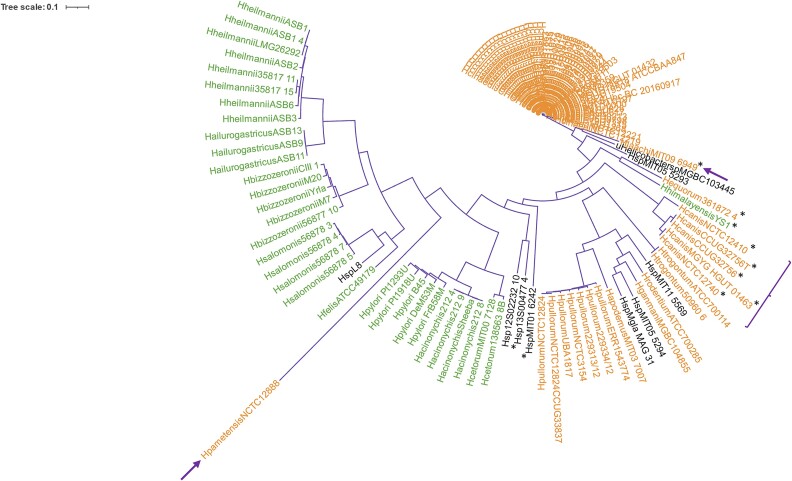
Hosts bacteria phylogenetic tree based on 181 orthologous gene sequences. Tree obtained using the FastTreeMP v2.1.11 program and visualized with software iTOL v5. Green indicates gastric species; orange, enterohepatic species; black, *Helicobacter* sp. The bold purple arrows and braces represent the hosts of the prophages that were in an opposite cluster.

A phylogenetic tree for the hosts of the prophages (Fig. 2) displays two main clusters: enterohepatic species and gastric species, similar to the prophage tree. *Helicobacter cinaedi* species are grouped closely together in the enterohepatic cluster. *Helicobacter pylori* species are located in the gastric cluster. Notably, *H. pametensis* (HpametensisNCTC12888), an enterohepatic species, appears in the gastric cluster. The gastric *H. himalayensis* is in the enterohepatic cluster, aligning with previous core genome studies [1]. *Helicobacter himalayensis* is evolutionarily close to *H. equorum* and *H. cinaedi* but not to other gastric species, which is in accordance with previous core genome study [43] of *Helicobacter* species. This particular strain *H. himalayensis* YS1 was isolated from the gastric mucosa from *Marmota himalayana*, the animal reservoir of *Yersinia pestis* in China. The phylogenetic analysis of 16S rRNA, 23S rRNA, and *gyrB* genes of *H. himalayensis* YS1 showed that the in all cases the sequences were more similar to other enterohepatic species [43]. *Helicobacter pametensis*, clustering with gastric species but on a long branch, indicates divergence from this group. When initially described, *H. pametensis* (isolated from a fecal sample of a gull) was found to be more closely related to *H. mustelae* (a gastric species) according to 16S rRNA gene [44]. These findings explain the unique positioning of *H. himalayensis* and *H. pametensis* in the phylogenetic tree.

An additional phylogenetic tree taking only the genomes of prophages from nine gastric species (*H. acinonychis*, *H. heilmannii*, *H. ailurogastricus*, *H. felis*, *H. salomonis*, *H. bizzozeronii*, *H. himalayensis*, *H. cetorum*, and *H. pylori*) showed that most prophages form well-defined clusters by host species, except for the *H. heilmannii* prophages that are polyphyletic, indicating that recombination and horizontal gene transfer in general may have given rise to the polyphyletic organization observed (Fig. S3). This indicates that the prophages of *H. heilmannii* do not share a single common ancestor exclusive to them and are distributed among multiple evolutionary lineages. Particularly, HheilmanniiASB3 shows substantial divergence, suggesting a longer evolutionary distance from other *H. heilmannii* prophages. The clusters of the prophages of *H. salomonis*, *H. pylori*, and *H. ailurogastricus* have relatively short branch lengths within their clades, indicating that the prophages within each group are closely related and share a recent common ancestor.

The gastric species tree for the bacterial hosts, constructed with 673 shared orthologous genes (Fig. S4), also displays the nine gastric species in different colors. As expected, all species form monophyletic clades, reporting to a common ancestor.

Identically, a phylogenetic tree was made for the genomes of the enterohepatic prophages (*H. cinaedi, H. pullorum, H. equorum, H. pametensis, H. jaachi, H. canis, H. ganmani, H. trogontum, H. rodentium*, and *H. apodemus*), revealing monophyletic clades for *H. cinaedi*, *H. canis*, and *H. trogontum*, indicating a single evolutionary origin for prophages within these species (Fig. S5). The Hcinaedi12219 prophage is placed as a distinct branch within the larger Hcinaedi clade, indicating a divergence from its closest relatives within the Hcinaedi group. The distinct branch suggests that Hcinaedi12219 might represent a unique lineage within the Hcinaedi species, potentially indicating different evolutionary pressures or a distinct ecological niche. The rest of the Hcinaedi prophages form several nested clades with much shorter branch lengths, indicating closer relatedness and more recent common ancestry. The *H. pullorum* prophages do not form a single clade, making this group polyphyletic. This suggests horizontal gene transfer and recombination events, which are common in prophage evolution. As expected, no polyphyletic groups are found for the enterohepatic species hosts tree constructed with the 264 shared orthologous genes (Fig. S6).

A partial analysis of 30 gastric and 30 enterohepatic prophages confirmed that phylogenetic patterns were consistent and not biased by sample size (data not shown).

### Network analysis of shared genes

**Figure 3:**
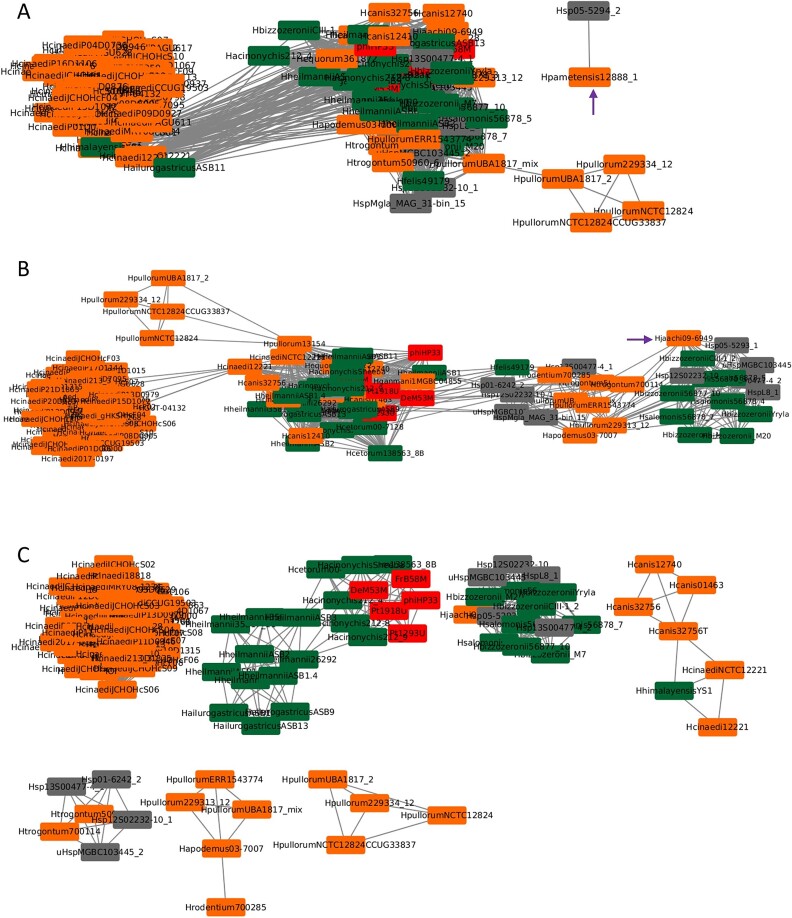
Network of orthologous genes for complete prophages of the *Helicobacter* genus. Orange indicates prophages from gastric species; green, prophages from enterohepatic species; red, *H. pylori* prophages; dark gray, not identified; purple arrows, enterohepatic prophages (*H. pameyensis* and *H. jaachi*) that were in the gastric cluster of the tree (Fig. 1). Orthologous genes, i.e. genes derived from a common ancestor by a speciation event, were determined using Proteinortho v.5.16 for all complete prophage sequences (*n* = 124: 119 prophages from non-*pylori Helicobacter* strains and 5 prophages from *H. pylori* strains) using a 25% identity and 50% coverage cut-off. A indicates at least 5 orthologous genes; B, at least 10 orthologous genes; C, at least 20 orthologous genes in common. Different numbers represent different groups.

In the network where at least five orthologous genes are present (Fig. 3A), each node represents a complete prophage, and a connection is present if the prophage sequences share five or more genes. There are two main groups: one is mostly composed of *H. cinaedi* complete prophages, having only one complete prophage from the gastric group (HhimalayensisYS1, which also pops out in the prophage genome phylogenetic trees), while the other group is composed of all the other complete prophages. There are two complete prophages (Hpametensis12888_1 and Hsp05_5294_2) that are not connected with neither one of the groups, being in agreement with Fig. 1, where the Hpamentensis12888 seems to be phylogenetically distant from the rest of the prophages and, despite the fact of it being a prophage from an enterohepatic species, is on the gastric cluster. Requiring at least 10 orthologous genes, five groups can be observed (Fig. 3B), being the gastric prophages present in two groups. Increasing the gene sharing for 20 orthologous genes, eight different groups emerge, being important to point that the majority of the gastric prophages are compressed within three of these groups in contrast with the enterohepatic groups, which show higher diversity. The gastric HimalayensisYS1 prophage (from a host species isolated from *Marmota*) is connected to enterohepatic prophages from *H. canis* and *H. cinaedi*, both isolated from dogs. This connection reinforces the sharing of prophage genes among distinct *Helicobacter* species (Fig. S7). The shared orthologous network and phylogenetic tree show that the closest prophages to *H. pylori* are the ones from *H. acynonichis* (isolated from big cats) and *H. cetorum* (isolated from beached Atlantic white-sided dolphin and captive whale).

### Phylogenetic congruence

Cophylogeny happens when two groups of phylogenies and interactions are in concordance, which can mean shared evolutionary history; although the process (evolutionary and biogeographical) that makes these results is not known [40]. The cophylogenetic signal between bacteria and prophage phylogenies (Figs 1 and 2) was measured using PACo [40], resulting in a *P*-value of 0 after 100 000 permutations, indicating dependence of prophage phylogeny on host phylogeny. In line, the ParaFitGlobal test [41] yielded a *P*-value of .001 after 999 permutations, also suggesting co-evolution between prophage and host bacteria. Among the 119 host and 124 prophage pairs, H0 was accepted in 10 cases (Table S5). This indicates that for these pairs the evolution is independent, i.e. the bacterial ordination does not predict prophage ordination, meaning prophage clades are randomly associated with bacterial host clades and their evolution has been independent. The pairs of prophage–bacteria whose evolution is independent include all *H. canis* prophages, as well as the prophages Hequorum361872, HhimalayensisYS1, Hjaachi09_6949, and several *Helicobacter* sp. prophages.

**Figure 4:**
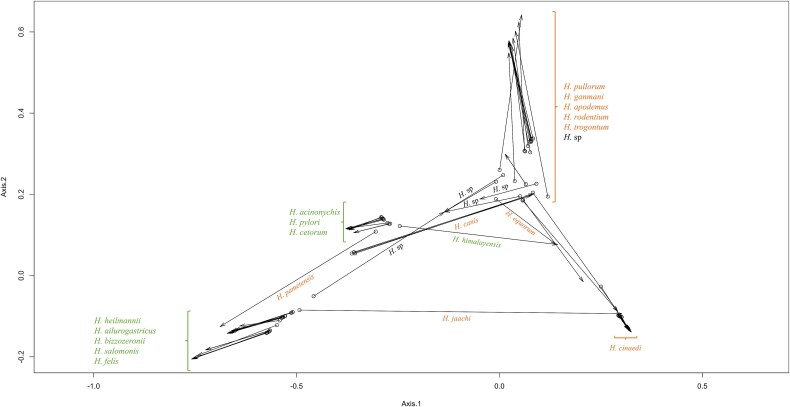
Procrustean superimposition plot for all complete prophages produced with PACo. In the arrows presented, the circle represents the parasite (prophage) and the tip of the arrow represents the host (bacteria). The projection of residuals onto the first two axes is given by the length of the arrow.

The Procrustean superimposition plot (Fig. 4) for all complete prophages and their hosts, created using PACo, displays the differences between prophage and host coordinates of patristic distances (measures evolutionary divergence between taxa). The plot reveals a clear division between gastric and enterohepatic prophage–host pairs, with exceptions: the *H. pametensis* pair (enterohepatic) within the gastric pairs; vice versa for the enterohepatic prophages of *H. canis* and *H. jaachi* pairs; and the *H. himalayensis* pair (gastric) with the prophage toward the gastric group side and the bacterial host on the enterohepatic side. These results align with the phylogenetic trees (Figs 1 and 2) and the network analysis of shared genes (Fig. 3). Given the fact that the length of the arrows is inversely proportional to the phylogenetic congruence based on the branching structure of the tree (topological)—meaning that the longer the length of the arrow, the less congruent is the pair [40]—the pairs pointed above are most likely to be the ones with less prophage–host congruence.

Likewise, the comparison of the phylogenetic trees for prophage–bacteria pairs using the gastric and enterohepatic phylogenetic trees provides a similar result, indicating significant overall co-evolution between prophages and bacteria (*P* < .001). However, the ParaFitGlobal results for the enterohepatic prophage bacteria pair of trees point to distinct co-evolution for the prophage Hjaachi09_6949 that evidences an independent evolution (H0 not rejected, p.F1 of 0.980, and p.F2 of 0.990).

### Synteny analysis

**Figure 5:**
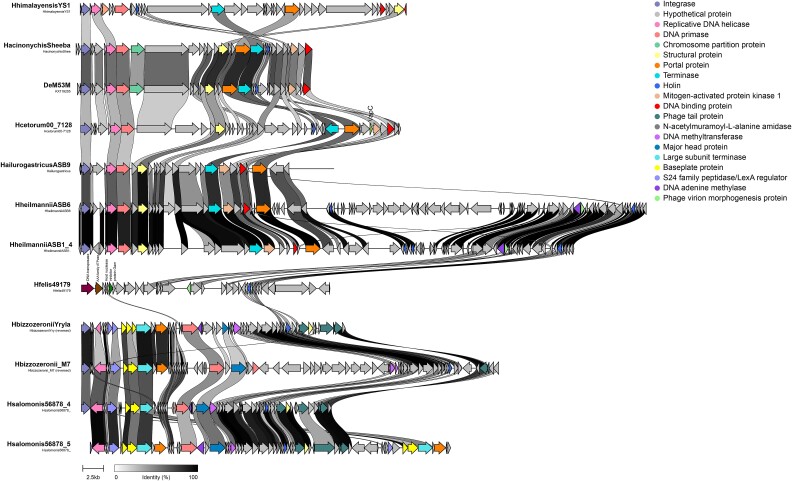
Biosynthetic gene cluster map of representative gastric species prophages. Figure obtained with clinker and clustermap.js. Arrows represent genes colored by function and connections represent similarity with a minimum alignment sequence identity of 0.3.

The gastric prophages have a quite similar gene order in relation to each other, with the exception of the Hfelis49179, i.e. different from the rest, making the order of the genes highly conserved even if it has some exceptions (Figs 5 and S8). This prophage uniquely includes genes for a DNA transposase, AAA ATPase, and a nuclease (Gam), all in its early sequence. Additionally, it has genes for a phage morphogenesis protein, a holin, and a lysin. Prophage HhimalayensisYS1, which stands out in both the phylogenetic tree and network analysis, also exhibits distinct gene content and organization. Notably, its DNA primase and DNA binding genes lack homology with similar genes in other gastric species prophages. Most gastric prophages start with an integrase, followed by replication and modification genes, regulatory elements, structural proteins, lysin, and holin. They include a DNA primase, which is sometimes positioned immediately after the helicase and other times further along the sequence. This conserved gene order suggests its role in phage cycle regulation. Large insertions of genes with unknown function can be found in prophages HheilmanniiASB6 and Hbizzozeronii_M7. These genes could potentially be considered cargo genes, yet their role remains challenging to determine due to the lack of comprehension regarding their functions.

**Figure 6:**
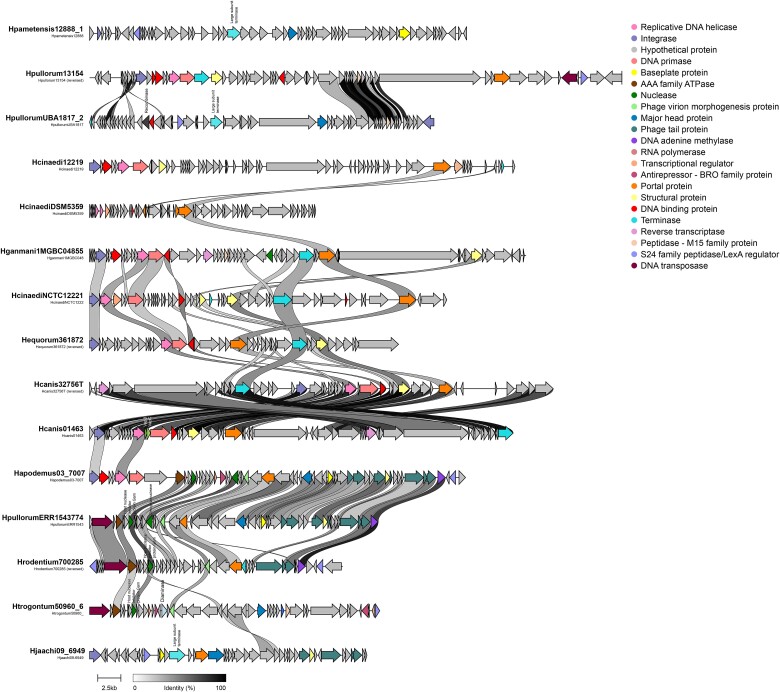
Biosynthetic gene cluster map of representative enterohepatic species prophages. Figure obtained with clinker and clustermap.js. Arrows represent genes colored by function and connections represent similarity with a minimum alignment sequence identity of 0.3.

Unlike the gastric species prophages, the prophages from the enterohepatic group are not that similar to each other, a fact that is congruent with what was previously seen in family determination and gene network analysis (Figs 6 and S8). Similar to gastric *Helicobacter* prophages, enterohepatic prophages with an integrase coding gene typically have this gene at the beginning of the sequences, followed by replication and modification genes, structural genes, and sometimes regulatory genes at the end.

## Discussion

Our analysis of prophage presence in species of the *Helicobacter* genus reveals their frequent occurrence, offering a comprehensive catalog that contrasts with the previously limited and sporadic reports. This finding highlights the broader relevance of prophages within these species and opens new avenues for understanding their evolutionary and functional roles.

There is a clear distinction in the distribution of complete and incomplete prophages across different species of *Helicobacter*. While in some species, like *H. cinaedi* and *H. felis*, most of the analyzed genomes exhibited a noteworthy number of complete or incomplete prophages, others within the analyzed group possessed only one prophage or none at all. This uneven distribution suggests a complex relationship between the host species and prophage acquisition, potentially influenced by evolutionary history, host environment, or microbial niche adaptation. The observation that certain species lack both complete and incomplete prophages suggests that these species may have diverged before acquiring prophages. It is also possible that these species occupy environments or possess genomic traits that limit or exclude the integration of prophages, pointing to ecological or evolutionary factors that influence prophage acquisition. However, a limitation of this study is that poorly represented species may show an absence of prophages due to the limited number of sequenced genomes available. As more genomes are analyzed, these species could reveal hidden prophages, highlighting the importance of expanding genomic studies to better understand prophage distribution patterns in *Helicobacter*.

Gastric prophages, originating from the same gastric environment, exhibit high synteny, meaning they have relatively similar gene orders. This similarity suggests that these prophages can be preserved, inherited from a common ancestor, and maintained throughout the years [45]. In contrast, enterohepatic prophages, which come from bacterial hosts isolated from various body organs, show greater diversity in their gene order and content. Specifically, 169 orthologous genes were identified in gastric prophages, while 275 orthologous genes were found in enterohepatic prophages. The classification by morphotype of prophages from *Helicobacter* species revealed that gastric prophages predominantly fall into the *Podoviridae* family (52%), indicating a commonality in their structure. The fact that a substantial proportion of enterohepatic prophages (83%) could not be classified further highlights the potential for unidentified prophage families and overall higher diversity. Orthologous genes sharing evaluated by network analysis showed that the enterohepatic *H. cinaedi* prophages form a cohesive group largely separated from other complete prophages. This finding underscores the high level of genetic similarity within *H. cinaedi* prophages, contrasting with the broader diversity observed in the other enterohepatic prophages. The presence of gastric prophages in multiple species and their compression into fewer clusters suggest a more conserved genetic structure within gastric environments compared to the more diverse enterohepatic prophages. This diversity among enterohepatic prophages likely reflects their adaptation to the distinct conditions of different body organs, demonstrating how varied environments can lead to greater genetic variability.

The mean prophage sequence length of ~30.8 Kb provides insight into the structural characteristics of the prophages. The relatively small size of these elements suggests that they might not carry extensive accessory genes but could still play a crucial role in bacterial evolution, virulence, or adaptability. Only 25.8% (123/476) of prophage orthologous genes had predicted functions, with few host-benefiting cargo genes identified, including a virulence gene, DNA methyltransferases, and type II toxin–antitoxin systems. These systems, which neutralize toxin effects to support prophage maintenance [46, 47], are also found in *H. pylori* prophages [12]. The lack of shared orthologous genes among all *Helicobacter* prophages is similar to their absence in other genera, such as *Desulfovibrio* [48].

The distinct GC content between the prophages and their host genomes (Table 2) suggests the potential involvement of horizontal gene transfer. This difference implies that the prophages likely originated from external sources and were integrated into the bacterial genomes rather than evolving together with the host from the beginning of species divergence. Notably, the GC% was not significantly different between *H. canis* and *H. pullorum*. While different GC content is often used as an indicator of foreign DNA [49], it does not always directly correspond to evolutionary relationships. Even with similar GC content, the differences in the phylogenetic trees of *H. canis* host and prophage reflect evolutionary divergence, highlighting that GC content alone is not sufficient to indicate co-evolution, underscoring the importance of multiple analyses to assess co-evolution. This may suggest that the prophages in *H. canis* were acquired recently from external sources with a similar GC content to the host genome.

The phylogenetic relationships between prophages and their bacterial hosts reveal complex interactions evidenced by the discrepancies found between the host and prophage trees. Similar to our findings in *Helicobacter*, a phylogenomic analysis of the core-genome sequences in the genus *Bifidobacterium* also revealed significant discrepancies when compared to the evolutionary development of prophage-like sequences [50]. The unusual clustering of *H. jaachi*, *H. canis*, and *H. himalayensis* prophages suggests that these prophages may have originated from horizontal gene transfer or other evolutionary processes that led them to diverge from their host species’ expected cluster. This could indicate substantial gene flow or recombination events influencing the phylogenetic distribution of prophages across different host species. For example, *H. himalayensis* prophages cluster with gastric species, while its bacterial host clusters with gastric enterohepatic, indicating divergent evolutionary paths. The unique positioning of *H. himalayensis* highlights the intricate and sometimes counterintuitive nature of host–phage evolution, influenced by specific ecological and evolutionary pressures. In both the gastric prophage and bacterial trees, *H. ailurogastricus*, *H. acinonychis*, *H. pylori*, *H. salomonis*, *H. bizzozeronii*, and *H. salomonis* are monophyletic, forming distinct clades. This suggests that these species have a well-defined evolutionary lineage both in terms of their prophages and their overall bacterial genomes. In contrast, although *H. heilmannii* strains are closely related in terms of their core genome, the prophages they harbor have different evolutionary histories. This could suggest frequent horizontal gene transfer or acquisition of different prophages by *H. heilmannii* strains over time. The discrepancies between prophage and bacterial phylogenies suggest that prophages can have a dynamic and varied evolutionary history compared to the more conserved bacterial genomes. The polyphyletic nature of some prophages, namely, *H. pullorum, H. cinaedi*, *and H. heilmannii*, indicates noteworthy horizontal gene transfer and multiple evolutionary origins, which is less apparent in the core bacterial genome trees. The greater variability in branch lengths within prophage clades, compared to bacterial clades, highlights the dynamic nature of prophage evolution, likely driven by horizontal gene transfer and recombination events. In contrast, the shorter branch lengths in the bacterial tree indicate a more stable genetic makeup, with less divergence among bacterial strains. This comparison underscores the broader genetic divergence and more dynamic evolution of prophages compared to the relatively stable bacterial genomes. Co-evolution between prophage and bacteria was present in most cases, except for the pairs of prophage–bacteria of *H. canis*, *H. equorum*, *H. himalayensis*, and *H. jaachi*. Should a prophage not demonstrate co-evolution with its bacterial host, its source could likely be attributed to external origins, such as horizontal gene transfer events from different bacteria or environments. Particularly notable is the case of *H. pylori*. The observed pattern in the bacterial and prophage phylogenetic trees shows that *H. pylori* prophages, along with those from *H. acinonychis* and *H. cetorum*, share a high number of genes. This suggests possible cross-species interactions or shared evolutionary pressures, potentially related to shared animal hosts or food chains.

In summary, our study reveals the extensive presence and varied distribution of prophages in *Helicobacter* species. Gastric prophages show structural consistency, while enterohepatic prophages exhibit significant diversity. These patterns suggest complex evolutionary dynamics, including potential horizontal gene transfer, highlighting the need for further genomic exploration to fully understand prophage roles and evolution.

## Supplementary Material

HelicobacterProphagesSupV7_ycaf017

TableS1_ycaf017

## Data Availability

The prophages identified have been submitted to NCBI GenBank repository, with the submission number ID 2868154.
