# Supplementary material for: Prophage dynamics in gastric and enterohepatic environments: unraveling ecological barriers and adaptive transitions
Source: ISME Commun. 2025 Feb 4;5(1):ycaf017. doi: 10.1093/ismeco/ycaf017 (PMC11840440; doi:10.1093/ismeco/ycaf017)
Supplement: HelicobacterProphagesSupV7_ycaf017 [file helicobacterprophagessupv7_ycaf017.pdf]

Supplementary material

Prophage dynamics in gastric and enterohepatic environments: unraveling ecological barriers and adaptive transitions

M. Proença, L. Tanoeiro, J.G. Fox, F.F. Vale

Table S2. Name given to the found complete prophages and information (strain and host where strain was found).

Prophage name	*Helicobacter* species and strain	*Helicobacter* animal host Common name
Hacinonychis212_9	*Helicobacter acinonychis* 212_9	Lion
HacinonychisSheeba	*Helicobacter acinonychis* strain Sheeba	Lion
Hacinonychis212_4	*Helicobacter acinonychis* strain 212_4	Felidae
Hacinonychis212_8	*Helicobacter acinonychis* strain 212_8	Felidae
HailurogastricusASB11	*Helicobacter ailurogastricus* strain ASB11	Cat
HailurogastricusASB13	*Helicobacter ailurogastricus* strain ASB13	Cat
HailurogastricusASB9	*Helicobacter ailurogastricus* strain ASB9	Cat
Hapodemus03_7007	*Helicobacter apodemus* MIT-03-7007	Rat
HbizzozeroniiCIII_1_2	*Helicobacter bizzozeronii* CIII-1	Human
Hbizzozeronii56877_10	*Helicobacter bizzozeronii* strain 56877_10	Dog
Hbizzozeronii_M20	*Helicobacter bizzozeronii* strain M20	Dog
Hbizzozeronii_M7	*Helicobacter bizzozeronii* strain M7	Dog
HbizzozeroniiYryla	*Helicobacter bizzozeronii* strain Yryla	Dog
Hcanis01463	*Helicobacter canis* MGYG-HGUT-01463	Human
Hcanis12740	*Helicobacter canis* NCTC 12740	Human
Hcanis32756	*Helicobacter canis strain* CCUG 32756	Dog
Hcanis32756T	*Helicobacter canis* strain CCUG 32756T	Dog
Hcanis12410	*Helicobacter canis* strain NCTC12410	Dog
Hcetorum00_7128	*Helicobacter cetorum* MIT 00-7128	Beluga whale
Hcetorum138563_8B	*Helicobacter cetorum* strain 138563_8B	Bottlenose dolphin
Hcinaedi18818	*Helicobacter cinaedi* CCUG 18818 = ATCC BAA-847	Human
HcinaediMRY08_1234	*Helicobacter cinaedi* DNA, strain: MRY08-1234	Human
Hcinaedi_gHKSHhc	*Helicobacter cinaedi* isolate gHKSHhc_BC_20160917	Human
Hcinaedi_MGYG_HGUT_04132	*Helicobacter cinaedi* MGYG-HGUT-01432	Human
HcinaediP01D00000	*Helicobacter cinaedi* P01D0000	Human
HcinaediP06D0798	*Helicobacter cinaedi* P06D0798	Human
HcinaediPAGU611	*Helicobacter cinaedi* PAGU611 DNA	Human
HcinaediPO3D0629	*Helicobacter cinaedi* PO3D0629	Human

Hcinaedi2017D_0197	*Helicobacter cinaedi* strain 2017D-0197	Human
Hcinaedi213_3	*Helicobacter cinaedi* strain 213_3	Human
HcinaediCCUG19503	*Helicobacter cinaedi* strain CCUG19503	Human
HcinaediCCUG19504	*Helicobacter cinaedi* strain CCUG19504	Human
HcinaediD7095	*Helicobacter cinaedi* strain D7095	Human
HcinaediDSM5359	*Helicobacter cinaedi* strain DSM 5359	Human
HcinaediJCHOHcF01	*Helicobacter cinaedi* strain JCHOHcF01	Human
HcinaediJCHOHcF02	*Helicobacter cinaedi* strain JCHOHcF02	Human
HcinaediJCHOHcF03	*Helicobacter cinaedi* strain JCHOHcF03	Human
HcinaediJCHOHcF04	*Helicobacter cinaedi* strain JCHOHcF04	Human
HcinaediJCHOHcF05	*Helicobacter cinaedi* strain JCHOHcF05	Human
HcinaediJCHOHcF06	*Helicobacter cinaedi* strain JCHOHcF06	Human
HcinaediJCHOHcF07	*Helicobacter cinaedi* strain JCHOHcF07	Human
HcinaediJCHOHcF08	*Helicobacter cinaedi* strain JCHOHcF08	Human
HcinaediJCHOHcF09	*Helicobacter cinaedi* strain JCHOHcF09	Human
HcinaediJCHOHcF10	*Helicobacter cinaedi* strain JCHOHcF10	Human
HcinaediJCHOHcS01	*Helicobacter cinaedi* strain JCHOHcS01	Human
HcinaediJCHOHcS02	*Helicobacter cinaedi* strain JCHOHcS02	Human
HcinaediJCHOHcS03	*Helicobacter cinaedi* strain JCHOHcS03	Human
HcinaediJCHOHcS04	*Helicobacter cinaedi* strain JCHOHcS04	Human
HcinaediJCHOHcS05	*Helicobacter cinaedi* strain JCHOHcS05	Human
HcinaediJCHOHcS06	*Helicobacter cinaedi* strain JCHOHcS06	Human
HcinaediJCHOHcS07	*Helicobacter cinaedi* strain JCHOHcS07	Human
HcinaediJCHOHcS08	*Helicobacter cinaedi* strain JCHOHcS08	Human
HcinaediJCHOHcS09	*Helicobacter cinaedi* strain JCHOHcS09	Human
HcinaediJCHOHcS10	*Helicobacter cinaedi* strain JCHOHcS10	Human
Hcinaedi12219	*Helicobacter cinaedi* strain NCTC12219	Dog
HcinaediNCTC12221	*Helicobacter cinaedi* strain NCTC12221	Dog
Hcinaedi12221	*Helicobacter cinaedi* strain NCTC12221	Dog
HcinaediP02D0213	*Helicobacter cinaedi* strain P02D0213	Human
HcinaediP04D0736	*Helicobacter cinaedi* strain P04D0736	Human
HcinaediP05D0741	*Helicobacter cinaedi* strain P05D0741	Human
HcinaediP07D0876	*Helicobacter cinaedi* strain P07D0876	Human
HcinaediP08D0905	*Helicobacter cinaedi* strain P08D0905	Human
HcinaediP09D0927	*Helicobacter cinaedi* strain P09D0927	Human
HcinaediP10D0937	*Helicobacter cinaedi* strain P10D0937	Human
HcinaediP11D0946	*Helicobacter cinaedi* strain P11D0946	Human
HcinaediP11D1015	*Helicobacter cinaedi* strain P11D1015	Human
HcinaediP12D0946	*Helicobacter cinaedi* strain P12D0946	Human
HcinaediP13D0979	*Helicobacter cinaedi* strain P13D0979	Human
HcinaediP14D1067	*Helicobacter cinaedi* strain P14D1067	Human

HcinaediP15D1072	*Helicobacter cinaedi* strain P15D1072	Human
HcinaediP16D1106	*Helicobacter cinaedi* strain P16D1106	Human
HcinaediP17D1144	*Helicobacter cinaedi* strain P17D1144	Human
HcinaediP18D1268	*Helicobacter cinaedi* strain P18D1268	Human
HcinaediP19D1315	*Helicobacter cinaedi* strain P19D1315	Human
HcinaediP20D1835	*Helicobacter cinaedi* strain P20D1835	Human
HcinaediP21D1863	*Helicobacter cinaedi* strain P21D1863	Human
HcinaediPAGU617	*Helicobacter cinaedi* strain PAGU617	Human
HcinaediPAGU628	*Helicobacter cinaedi* strain PAGU628	Human
Hequorum361872	*Helicobacter equorum* strain 361872_4	Pig
Hfelis49179	*Helicobacter felis* ATCC 49179	Cat
Hganmani1MGBC04855	*Helicobacter ganmani* strain MGBC104855	Mouse
HheilmanniiASB1_4	*Helicobacter heilmanii* ASB1.4	Cat
Hheilmannii35817_11	*Helicobacter heilmannii* strain 35817_11	Cat
Hheilmannii35817_15	*Helicobacter heilmannii* strain 35817_15	Cat
HheilmanniiASB1	*Helicobacter heilmannii* strain ASB1	Cat
HheilmanniiASB2	*Helicobacter heilmannii* strain ASB2	Cat
HheilmanniiASB3	*Helicobacter heilmannii* strain ASB3	Cat
HheilmanniiASB6	*Helicobacter heilmannii* strain ASB6	Cat
Hheilmannii26292	*Helicobacter heilmannii* strain LMG 26292	Cat
HhimalayensisYS1	*Helicobacter himalayensis* YS1	Marmota
Hjaachi09_6949	*Helicobacter jaachi* MIT 09-6949	Marmoset
Hpametensis12888_1	*Helicobacter pametensis* strain NCTC12888	Gull
HpullorumNCTC12824	*Helicobacter pullorum* NCTC 12824	Chicken
HpullorumNCTC12824CCUG33837	*Helicobacter pullorum* NCTC 12824 strain CCUG 33837	Chicken
Hpullorum229313_12	*Helicobacter pullorum* strain 229313/12	Chicken
Hpullorum229334_12	*Helicobacter pullorum* strain 229334/12	Chicken
HpullorumERR1543774	*Helicobacter pullorum* strain ERR1543774_bin.1_metaWRAP_v1.1_MAG	Chicken
Hpullorum13154	*Helicobacter pullorum* strain NCTC13154	Chicken
HpullorumUBA1817_mix	*Helicobacter pullorum* strain UBA1817	Chicken
HpullorumUBA1817_2	*Helicobacter pullorum* strain UBA1817	Chicken
Hrodentium700285	*Helicobacter rodentium* ATCC 700285	Mouse
Hsalomonis56878_3	*Helicobacter salomonis* strain 56878_3	Dog
Hsalomonis56878_4	*Helicobacter salomonis* strain 56878_4	Dog
Hsalomonis56878_5	*Helicobacter salomonis* strain 56878_5	Dog
Hsalomonis56878_7	*Helicobacter salomonis* strain 56878_7	Dog
Hsp12S02232_10	*Helicobacter* sp. 12S02232-10	Rhinoceros iguana
Hsp12S02232_10_1	*Helicobacter* sp. 12S02232-10	Rhinoceros iguana
Hsp13S00477_4_1	*Helicobacter* sp. 13S00477-4	House gecko

Hsp13S00477_4_2	*Helicobacter* sp. 13S00477-4	House gecko
HspL8_1	*Helicobacter* sp. L8	Red fox
Hsp01_6242_2	*Helicobacter* sp. MIT 01-6242	Sea otter
Hsp05_5293_1	*Helicobacter* sp. MIT 05-5293	Mouse
Hsp05_5294_2	*Helicobacter* sp. MIT 05-5294	Mouse
Hsp11_5569	*Helicobacter* sp. MIT 11-5569	Marmoset
HspMgla_MAG_31_bin_15	*Helicobacter* sp. strain Mgla_MAG_31-bin_15	Bank vole
Htrogontum700114	*Helicobacter trogontum* ATCC 700114	Rat
Htrogontum50960_6	*Helicobacter trogontum* strain 50960_6	Rat
uHspMGBC103445	uncultured *Helicobacter* sp. strain MGBC103445	Mouse
uHspMGBC103445_2	uncultured *Helicobacter* sp. strain MGBC103445	Mouse

Table S3. Virfam results. Species, family, type, cluster and prophages of the species with that classification.

Species	Family	Type	Cluster	Prophages
H. acinonychis	*Podoviridae*	Type 3		All
H. ailurogastricus	*Podoviridae*	Type 3		All
H. apodemus	*Myoviridae*	Type 1		All
H. bizzozeronii	*Siphoviridae*			Hbizzozeronii_M7
	Myoviridae			The rest
H. canis	None			All
H. cetorum	*Podoviridae*	Type 3		All
H. cinaedi	*Podoviridae*	Type 3		Hcinaedi12219; Hcinaedi12221
	None			The rest
H. equorum	None			All
H. felis	*Siphoviridae*			All
H. ganmani	*Podoviridae*	Type 3		All
H. heilmannii	Hybrid *Siphoviridae* *Podoviridae*	no type Type 3		The rest
	Podoviridae	Type 3		HheilmanniiASB1.4; HheilmanniiASB3
H. himalayensis	*Podoviridae*	Type 3		All
H. jaachi	*Myoviridae*	Type 1	Cluster 6	All
H. pametensis	*Myoviridae*	Type 1	Cluster 7	All
H. pullorum	*No results*			Hpullorum13154
	Myoviridae	Type 1	Cluster 8	Hpullorum229313_12, HpullorumERR1543774; HpullorumUBA1817_mix
	None			The rest
H. pylori	*Podoviridae*	Type 3		All
H. rodentium	*Siphoviridae*			All
H. salomonis	*Myoviridae*			All
Helicobacter sp.	*Myoviridae*	Type 1	Cluster 3	Hsp05_5293_1
			Cluster 7	Hsp05_5294_2
				Hsp12S02232_10; Hsp13S00477_4_2; HspL8_1
	Podoviridae	Type 3		Hsp11_5569
	Siphoviridae			Hsp01_6242_2; Hsp12S02232_10_1; Hsp13S00477_4_1; HspMgla_MAG_31_bin_15
H. trogontum	*Siphoviridae*			All
unc. *Helicobacter* sp.	*Myoviridae*	Type1	Cluster 3	uHspMGBC103445
	Siphoviridae			uHspMGBC103445_2

Table S4. Orthologous genes found and number of prophages where they were found.

Orthologous genes/Predicted protein function	Number prophage sequences
Portal protein	84
DNA binding protein	61
Transcriptional regulator	58
Transcriptional regulator	56
DNA helicase	55
Replicative DNA helicase	55
Integrase	50
Integrase	48
Portal protein	44
DNA primase	41
Portal protein	40
Holin	36
Major head protein	34
Transcriptional regulator	34
Terminase	33
Replicative DNA helicase	33
Tail fiber protein	29
Tail assembly chaperone	27
Mitogen-activated protein kinase 1	23
DNA adenine methylase	21
Phage tail protein	21
Tail length tape measure protein	19
N-acetilmuramoyl-L-alanine amidase	18
Tail completion Neck1 protein	18
Baseplate protein	18
Tail sheath	18
Minor head protein and DNA pilot	18
Baseplate wedge subunit	18
Head closure	18
Baseplate protein	17
Phage tail protein	15
Large subunit terminase	14
Major head protein	14
Portal protein	13
AAA family atpase	13
Host nuclease inhibitor protein Gam	13
DNA transposase	13
Virion structural protein	13
Portal protein	13
Toxin	11
Regulator of chromosome condensation	11
Replication initiation protein	11
Terminase large subunit	11

Head maturation protease	11
CI like repressor	10
Chromosome partition protein	10
Virion structural protein	10
Nucleoside 2-deoxyribosyltransferase	10
DNA methyltransferase	8
Antirepressor - BRO family protein	8
XRE family transcriptional regulator	8
Peptidase - M15 family protein	8
S24 family peptidase/lexa regulator	7
Replicative DNA helicase	7
Major head protein	7
Replication initiation protein	7
Anti-repressor Ant	7
Portal protein	6
Phage tail protein	6
Transposase	6
Baseplate hub	6
Single-stranded DNA binding protein	5
Reverse transcriptase	5
Toxin-antitoxin system hicb-like	5
Toxin	5
Replication initiation protein	5
Holin	5
Replication initiation protein	5
Tail length tape measure protein	5
DNA primase	4
Major head protein	4
Major head protein	4
Phage tail protein	4
Deoxyribonuclease	4
Tail length tape measure protein	4
Exonuclease	4
Head-tail adaptor	4
Tail fiber protein	4
Virion structural protein	4
DNA binding protein	4
DNA helicase	4
Segregation and condensation complex subunit scpb	4
Transcriptional repressor	4
DNA methyltransferase	3
Rect like ssdna annealing protein	3
Large subunit terminase	3
Large subunit terminase	3
Trasposase	3
Integrase	3

Phage tail protein	3
Phage tail protein	3
RNA polymerase	3
Major head protein	3
Type II toxin-antitoxin system vapc family toxin	3
Type II toxin-antitoxin system vapb family toxin	3
Replication initiation protein	3
Major head protein	3
Endolysin	3
Rusa-like Holliday junction resolvase	3
DNA helicase	3
CI-like repressor	3
S24 family peptidase/lexa regulator	2
DNA primase	2
Transcriptional regulator - irre protein	2
Dna methyltransferase	2
DNA helicase	2
Integrase	2
Recombinase/integrasse	2
Major head protein	2
Baseplate protein	2
Large subunit terminasse	2
Terminase	2
Virulence associated protein	2
Dctp diaminase	2
Terminase	2
Transposase	2
Transcriptional repressor	2
Tail assembly chaperone	2
Tail completion or Neck1 protein	2
Portal protein	2
Transcriptional repressor	2
Transcriptional regulator	2
Nucleotide kinase	2

Note: Repeated names indicate that the same function is predicted for sequences where the sequence identity falls below the default identity cutoff of Proteinortho v5.16.
Most of the genes coding for the same function were identified in prophages from different *Helicobacter* species. Exceptions, where genes with the same function were found within prophages of the same species, corresponded to sequences of different lengths that grouped separately based on the cutoffs used.

Table S5. Results of the test of individual host-parasite links from ParaFit for all complete prophages. F1.stat (value where trace is the ParaFitGlobal statistic), F2.stat (where the tracemax is the maximum value that can be taken by trace), p.F1 (p-value of F1.stat) and p.F2 (p-value of F2.stat). Prophage-bacteria pairs not in co-evolution are highlighted – in orange the gastric species, in green the hepatic species. Non-classified prophage-bacteria pairs not in co-evolution are highlighted in grey.

Prophage	F1.stat	p.F1	F2.stat	p.F2
Hacinonychis212_4	18.1198032	0.001	0.0083464879	0.001
Hacinonychis212_8	18.1038554	0.001	0.0083391419	0.001
Hacinonychis212_9	18.0947854	0.001	0.0083349640	0.001
HacinonychisSheeba	18.1249808	0.001	0.0083488728	0.001
HailurogastricusASB11	42.6216636	0.001	0.0196327297	0.001
HailurogastricusASB13	42.6559117	0.001	0.0196485053	0.001
HailurogastricusASB9	42.6757093	0.001	0.0196576247	0.001
Hapodemus09_7007	4.8554568	0.001	0.0022365591	0.001
Hbizzozeronii56877_10	35.7710367	0.001	0.0164771395	0.001
HbizzozeroniiCIII_1_2	35.2982224	0.001	0.0162593480	0.001
Hbizzozeronii_M20	35.4256272	0.001	0.0163180342	0.001
Hbizzozeronii_M7	36.1934930	0.001	0.0166717347	0.001
HbizzozeroniiYryla	35.0670336	0.001	0.0161528559	0.001
Hcanis32756	1.9781801	0.196	0.0009112050	0.107
Hcanis32756T	1.9781239	0.202	0.0009111792	0.116
Hcanis01463	2.1056801	0.185	0.0009699351	0.096
Hcanis12410	1.9871311	0.198	0.0009153281	0.097
Hcanis12740	2.1056801	0.182	0.0009699351	0.098
Hcetorum138563_8B	15.0412963	0.001	0.0069284415	0.001
Hcetorum00_7128	14.8277851	0.001	0.0068300923	0.001
Hcinaedi2017D_0197	10.3118141	0.001	0.0047499098	0.001
Hcinaedi213_3	10.2823283	0.001	0.0047363278	0.001
Hcinaedi18818	10.3643518	0.001	0.0047741102	0.001
HcinaediCCUG19503	10.1812721	0.001	0.0046897785	0.001
HcinaediCCUG19504	10.2733380	0.001	0.0047321867	0.001
HcinaediD7095	10.2938553	0.001	0.0047416375	0.001
HcinaediDSM5359	10.3322657	0.001	0.0047593304	0.001
Hcinaedi_gHKSHhc	10.3351337	0.001	0.0047606515	0.001
HcinaediJCHOHcF01	10.3000186	0.001	0.0047444765	0.001
HcinaediJCHOHcF02	10.3409224	0.001	0.0047633180	0.001
HcinaediJCHOHcF03	10.3204726	0.001	0.0047538982	0.001
HcinaediJCHOHcF04	10.3409254	0.001	0.0047633193	0.001
HcinaediJCHOHcF05	10.3410264	0.001	0.0047633658	0.001
HcinaediJCHOHcF06	10.4229128	0.001	0.0048010850	0.001
HcinaediJCHOHcF07	10.3819736	0.001	0.0047822273	0.001
HcinaediJCHOHcF08	10.3409978	0.001	0.0047633527	0.001
HcinaediJCHOHcF09	10.3205482	0.001	0.0047539330	0.001
HcinaediJCHOHcF10	10.3615005	0.001	0.0047727968	0.001
HcinaediJCHOHcS01	10.3409978	0.001	0.0047633527	0.001
HcinaediJCHOHcS02	10.3409978	0.001	0.0047633527	0.001
HcinaediJCHOHcS03	10.2590542	0.001	0.0047256071	0.001
HcinaediJCHOHcS04	10.3409978	0.001	0.0047633527	0.001
HcinaediJCHOHcS05	10.2590542	0.001	0.0047256071	0.001
HcinaediJCHOHcS06	10.4024461	0.001	0.0047916575	0.001
HcinaediJCHOHcS07	10.2795918	0.001	0.0047350674	0.001
HcinaediJCHOHcS08	10.2795636	0.001	0.0047350544	0.001
HcinaediJCHOHcS09	10.4229128	0.001	0.0048010850	0.001
HcinaediJCHOHcS10	10.4024431	0.001	0.0047916561	0.001
Hcinaedi_MGYG_HGUT_04132	10.3643518	0.001	0.0047741102	0.001
HcinaediMRY08_1234	10.2181129	0.001	0.0047067485	0.001
Hcinaedi12219	8.7607249	0.001	0.0040354348	0.001
Hcinaedi12221	10.5191153	0.001	0.0048453986	0.001
HcinaediNCTC12221	10.5994906	0.001	0.0048824217	0.001
HcinaediP01D0000	10.3234771	0.001	0.0047552821	0.001
HcinaediP02D0213	10.2381934	0.001	0.0047159981	0.001
HcinaediP03D0629	10.2590600	0.001	0.0047256098	0.001
HcinaediP04D0736	10.3204897	0.001	0.0047539060	0.001

HcinaediP05D0741	10.4023596	0.001	0.0047916176	0.001
HcinaediP06D0798	10.2694780	0.001	0.0047304086	0.001
HcinaediP07D0876	10.3818969	0.001	0.0047821919	0.001
HcinaediP08D0905	10.3409619	0.001	0.0047633361	0.001
HcinaediP09D0927	10.4228289	0.001	0.0048010464	0.001
HcinaediP10D0937	10.3818994	0.001	0.0047821931	0.001
HcinaediP11D0946	10.3086191	0.001	0.0047484381	0.001
HcinaediP11D1015	10.2999924	0.001	0.0047444644	0.001
HcinaediP12D0946	10.3000164	0.001	0.0047444755	0.001
HcinaediP13D0979	10.3785279	0.001	0.0047806401	0.001
HcinaediP14D1067	10.4023596	0.001	0.0047916176	0.001
HcinaediP15D1072	10.2385828	0.001	0.0047161774	0.001
HcinaediP16D1106	10.3204417	0.001	0.0047538840	0.001
HcinaediP17D1144	10.4108848	0.001	0.0047955445	0.001
HcinaediP18D1268	10.2900398	0.001	0.0047398800	0.001
HcinaediP19D1315	10.2900398	0.001	0.0047398800	0.001
HcinaediP20D1835	10.2385327	0.001	0.0047161544	0.001
HcinaediP21D1863	10.2385327	0.001	0.0047161544	0.001
HcinaediPAGU611	10.1812721	0.001	0.0046897785	0.001
HcinaediPAGU617	10.3440490	0.001	0.0047647581	0.001
HcinaediPAGU628	10.3440490	0.001	0.0047647581	0.001
Hequorum361872	0.5984904	0.284	0.0002756814	0.149
Hfelis49179	35.5773642	0.001	0.0163879285	0.001
Hganmani1MGBC04855	3.4349647	0.003	0.0015822407	0.001
Hheilmannii35817_11	44.9824094	0.001	0.0207201552	0.001
Hheilmannii35817_15	44.9615593	0.001	0.0207105511	0.001
HheilmanniiASB1	45.3616851	0.001	0.0208948602	0.001
HheilmanniiASB1.4	45.4244898	0.001	0.0209237898	0.001
HheilmanniiASB2	45.3025734	0.001	0.0208676317	0.001
HheilmanniiASB3	42.6984855	0.001	0.0196681160	0.001
HheilmanniiASB6	45.1490609	0.001	0.0207969195	0.001
Hheilmannii26292	45.3479919	0.001	0.0208885528	0.001
HhimalayensisYS1	-2.7717692	0.979	-0.0012767544	0.995
Hjaachi09_6949	-15.7450307	1.000	-0.0072526013	1.000
Hpametensis12888_1	24.5870907	0.001	0.0113255013	0.001
Hpullorum229313_12	5.4825103	0.001	0.0025253975	0.001
Hpullorum229334_12	5.7253359	0.001	0.0026372498	0.001
HpullorumERR1543774	5.4354736	0.001	0.0025037311	0.001
HpullorumNCTC12824	5.6308114	0.001	0.0025937091	0.001
HpullorumNCTC12824CCUG33837	5.6307554	0.001	0.0025936833	0.001
Hpullorum13154	5.1196699	0.001	0.0023582631	0.001
HpullorumUBA1817_2	5.5855679	0.001	0.0025728687	0.001
HpullorumUBA1817_mix	5.3866913	0.001	0.0024812606	0.001
DeM53M	17.8469957	0.001	0.0082208251	0.001
FrB58M	18.0224061	0.001	0.0083016241	0.001
phiHP33	18.5515614	0.001	0.0085453678	0.001
Pt1293U	18.2068505	0.001	0.0083865843	0.001
Pt1918U	17.2087240	0.001	0.0079268193	0.001
Hrodentium700285	5.1410504	0.001	0.0023681115	0.001
Hsalomonis56878_7	37.8355103	0.001	0.0174280937	0.001
Hsalomonis56878_3	37.7631086	0.001	0.0173947434	0.001
Hsalomonis56878_4	37.6522956	0.001	0.0173436999	0.001
Hsalomonis56878_5	37.7229089	0.001	0.0173762264	0.001
Hsp12S02232_10	1.9474377	0.039	0.0008970442	0.015
Hsp12S02232_10_1	6.4405795	0.014	0.0029667109	0.004
Hsp13S00477_4_1	0.5088292	0.285	0.0002343810	0.179
Hsp13S00477_4_2	1.8638514	0.060	0.0008585421	0.023
HspL8_1	36.6427015	0.001	0.0168786526	0.001
HspMgla_MAG_31_bin_15	5.3301407	0.001	0.0024552118	0.001
Hsp01_6242_2	1.2154231	0.143	0.0005598579	0.066
Hsp05_5293_1	1.2987905	0.076	0.0005982592	0.034
Hsp05_5294_2	4.0952035	0.002	0.0018863652	0.001
Hsp11_5569	3.3460440	0.002	0.0015412814	0.001

Htrogontum50960_6	2.8120432	0.010	0.0012953057	0.002
Htrogontum700114	2.8120838	0.012	0.0012953244	0.004
uHspMGBC103445	1.2910848	0.067	0.0005947097	0.034
uHspMGBC103445_2	2.1079062	0.024	0.0009709605	0.009

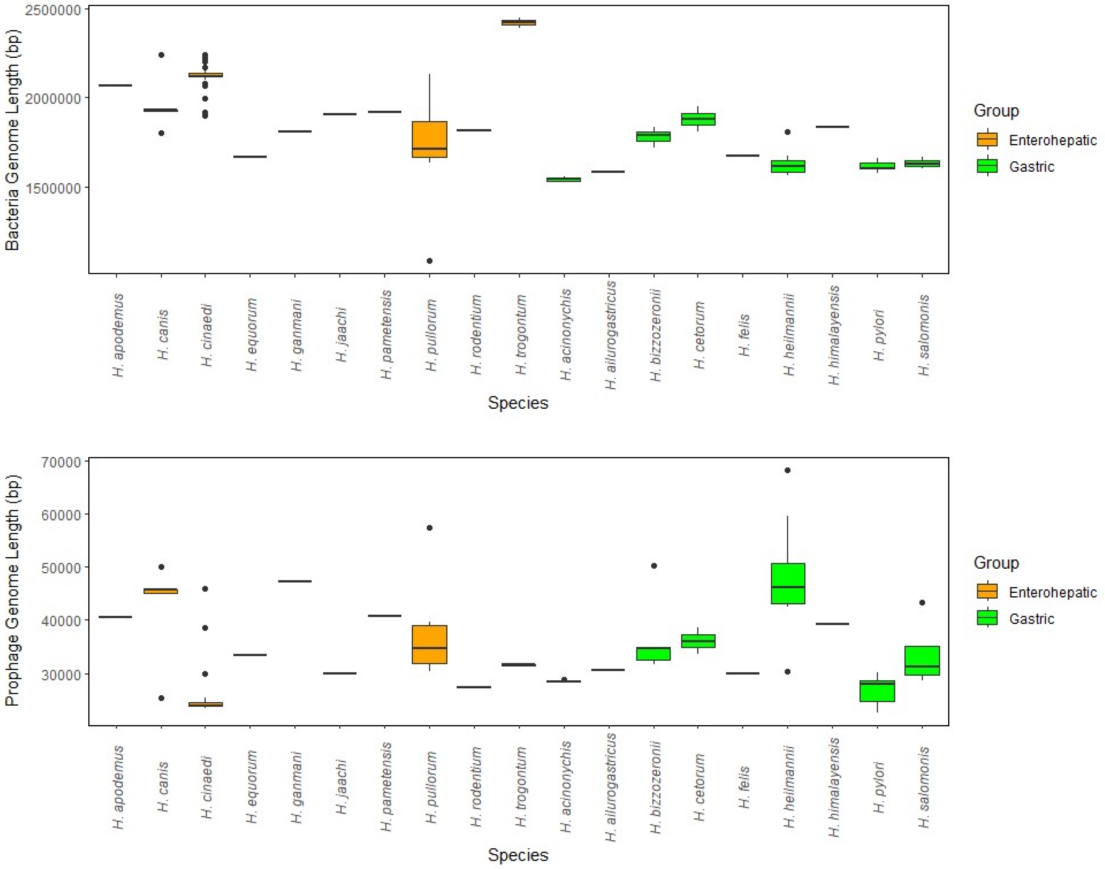

Figure S1. Box-plot of the host bacteria and prophage genome length sorted by *Helicobacter* species.

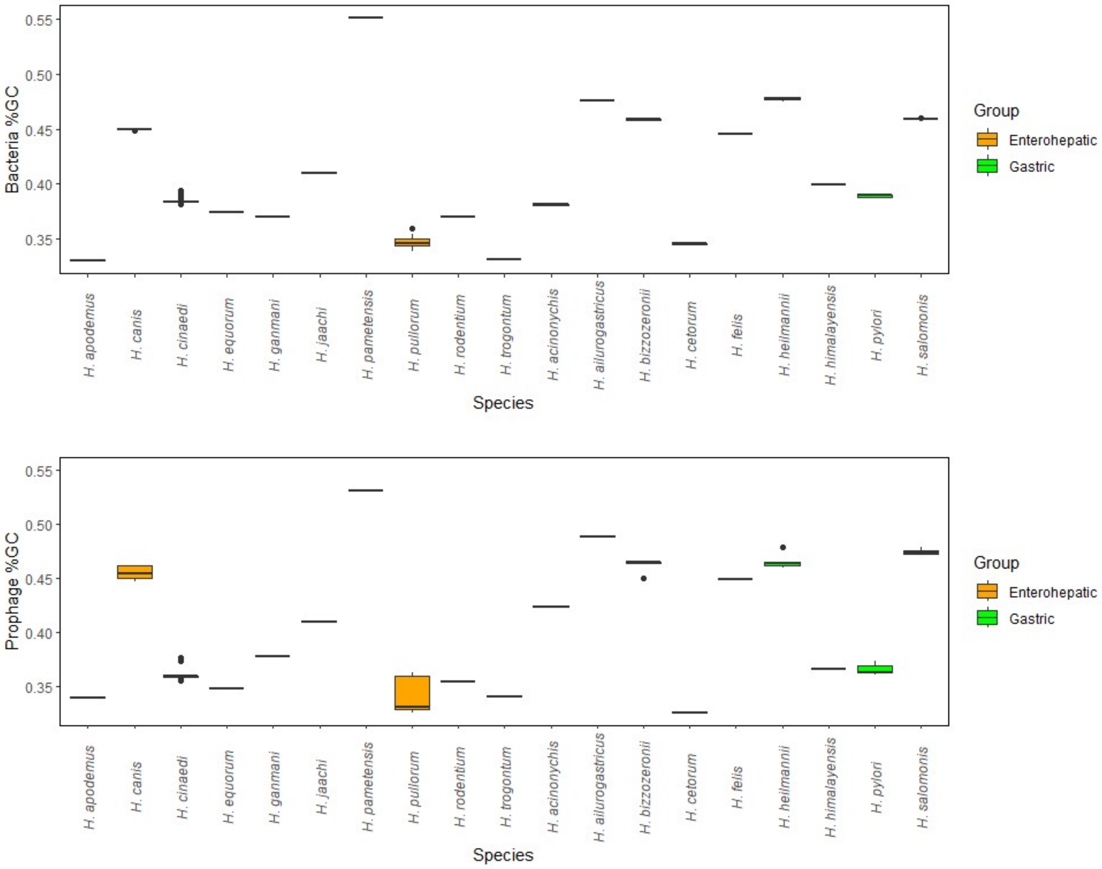

Figure S2. Box-plot of the host bacteria and prophage percentage of GC content sorted by *Helicobacter* species. %GC – percentage of guanine (G) and cytosine (C).

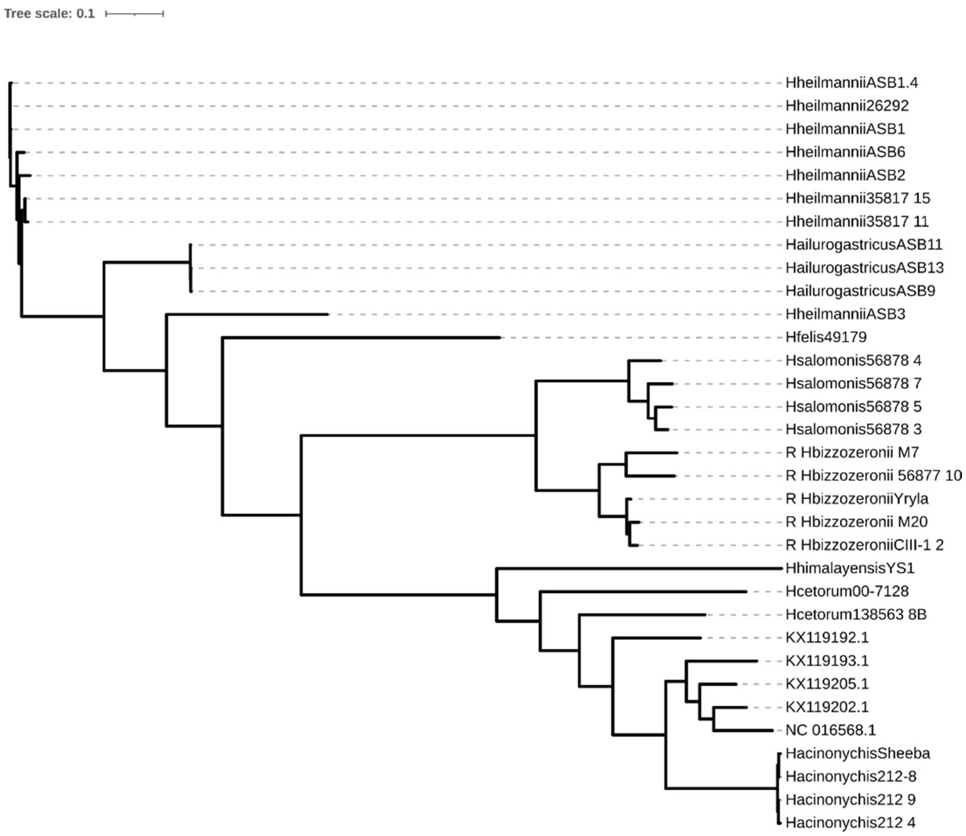

Figure S3. Phylogenetic tree of the complete genome prophages of the *Helicobacter* genus gastric species. Tree obtained using the FastTreeMP v2.1.11 program and visualized with software iTOL v5.

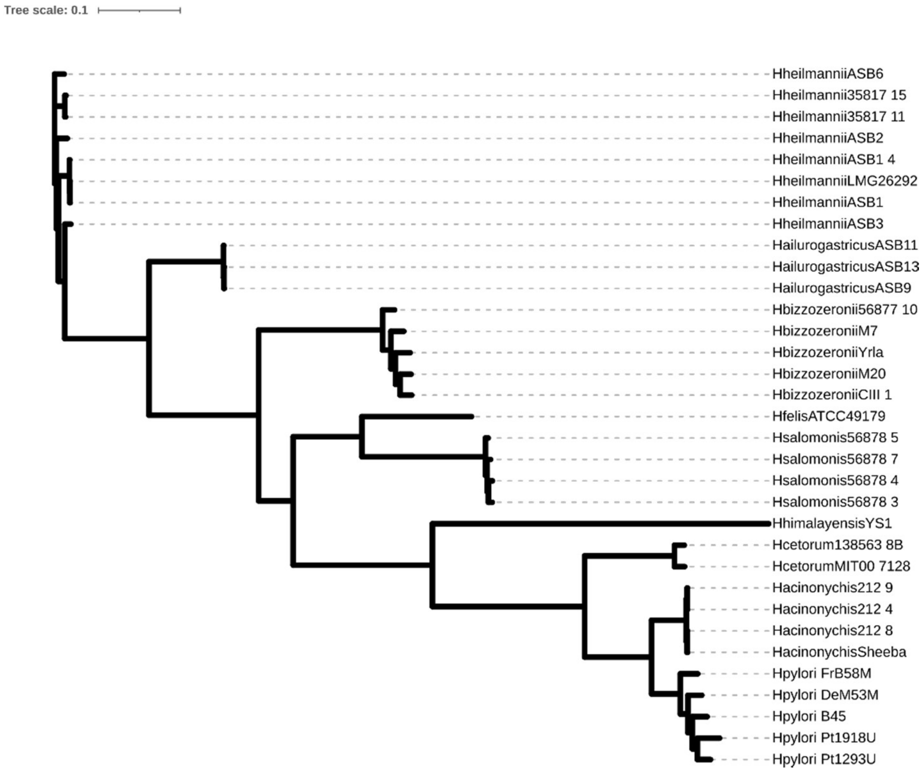

Figure S4. Phylogenetic tree of the 673 orthologous genes of the gastric bacterial hosts. Tree obtained using the FastTreeMP v2.1.11 program and visualized with software iTOL v5.

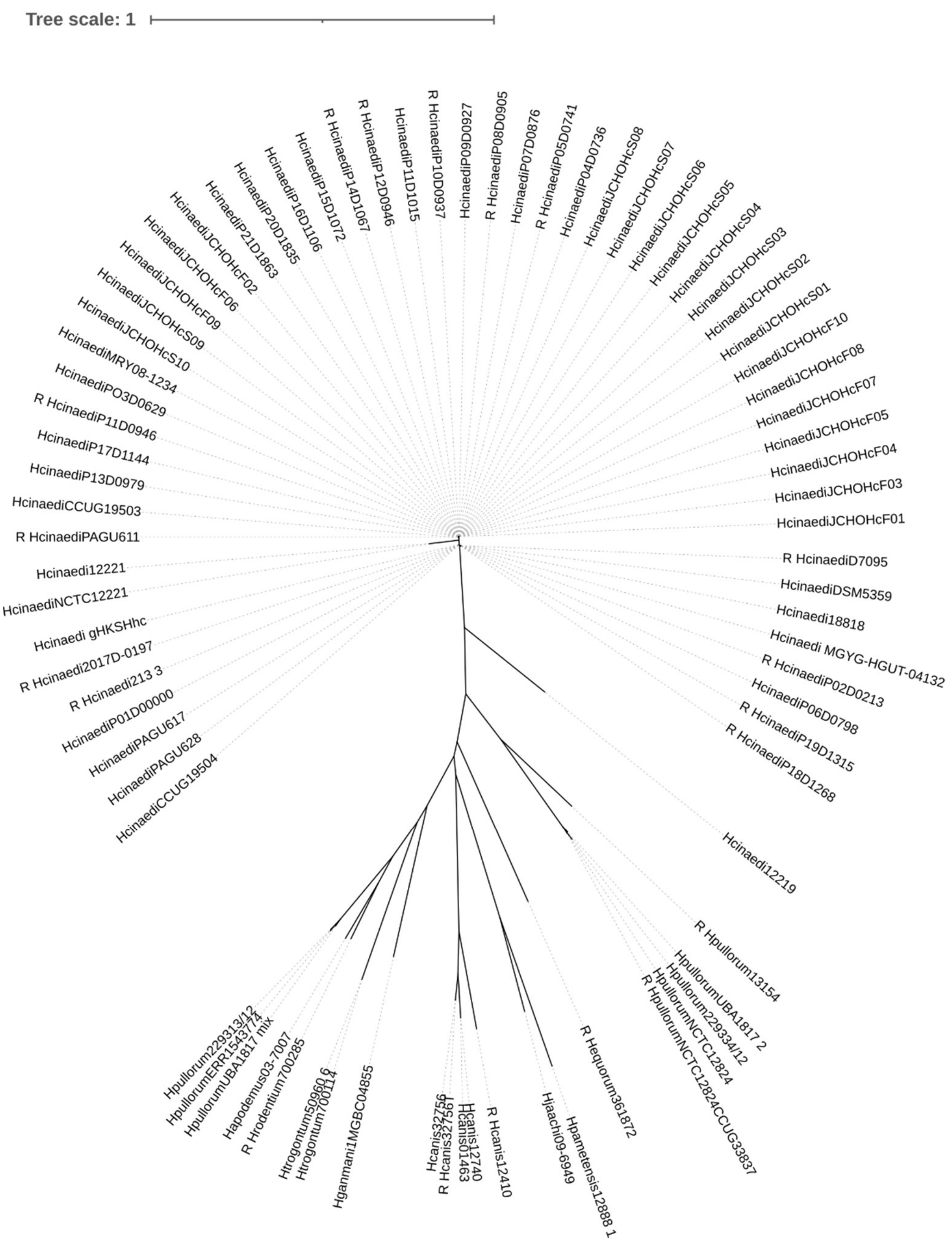

Figure S5. Phylogenetic tree of the complete genome prophages of the *Helicobacter* genus enterohepatic species. Tree obtained using the FastTreeMP v2.1.11 program and visualized with software iTOL v5.

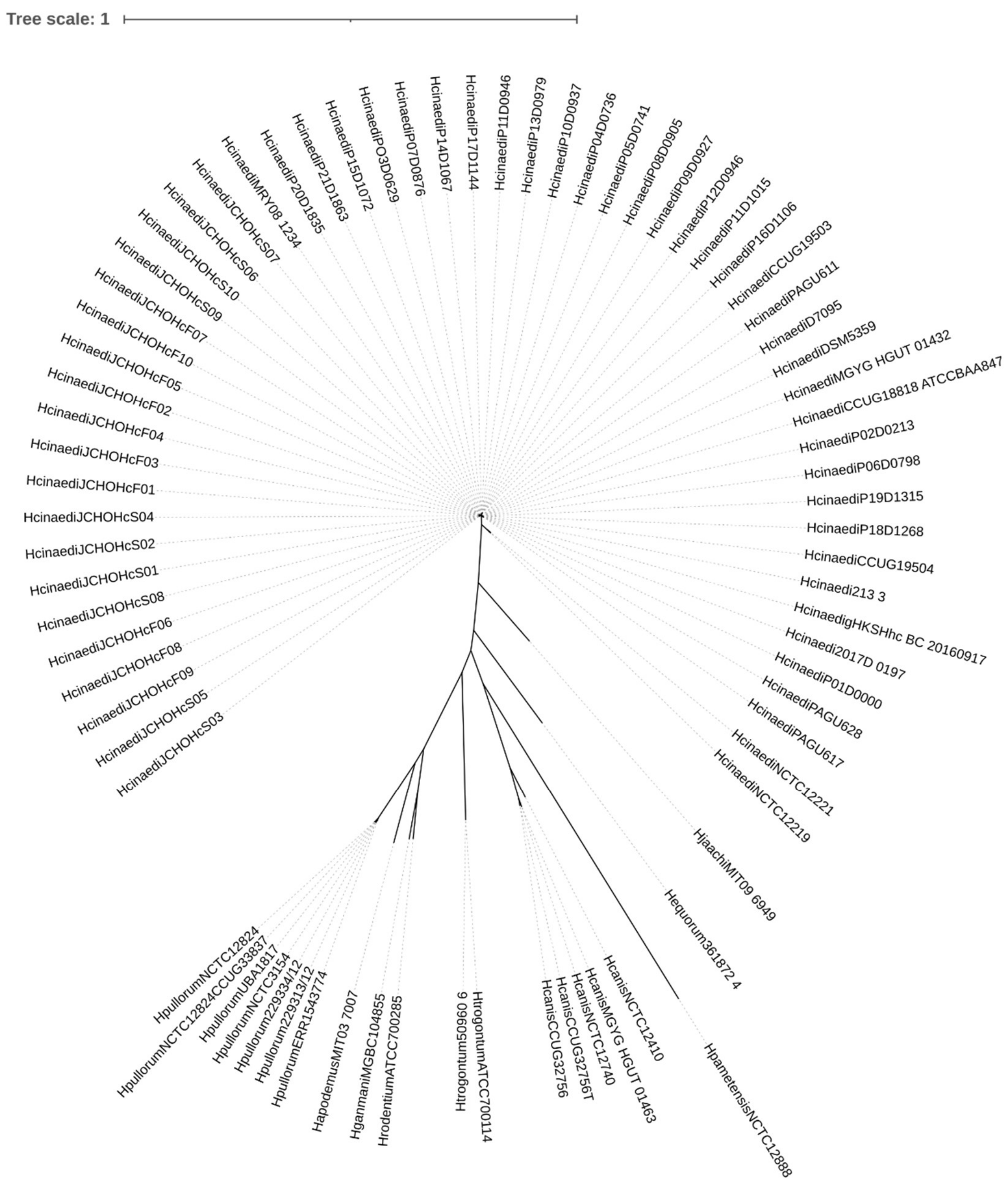

Figure S6. Phylogenetic tree of the 264 orthologous genes of the enteric bacterial hosts. Tree obtained using the FastTreeMP v2.1.11 program and visualized with software iTOL v5.

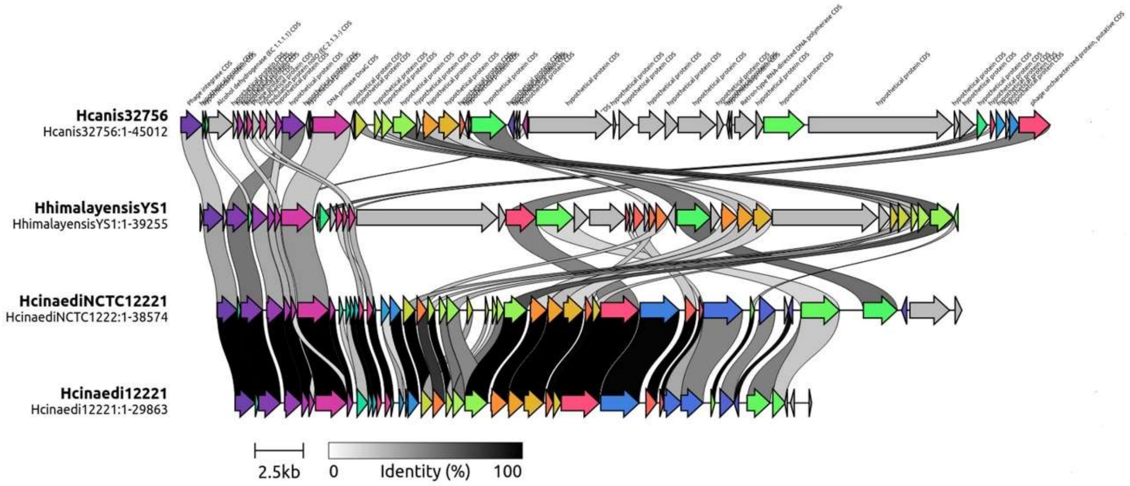

Figure S7. Biosynthetic gene cluster map of gastric HhimalayensisYS1 prophage (isolated from a marmota), showing its connection to enterohepatic prophages from *H. canis* and *H. cinaedi*, both isolated from dogs. Figure obtained with clinker and clustermap.js. Arrows represent genes colored by function, and connections represent similarity with minimum alignment sequence identity of 0.25.

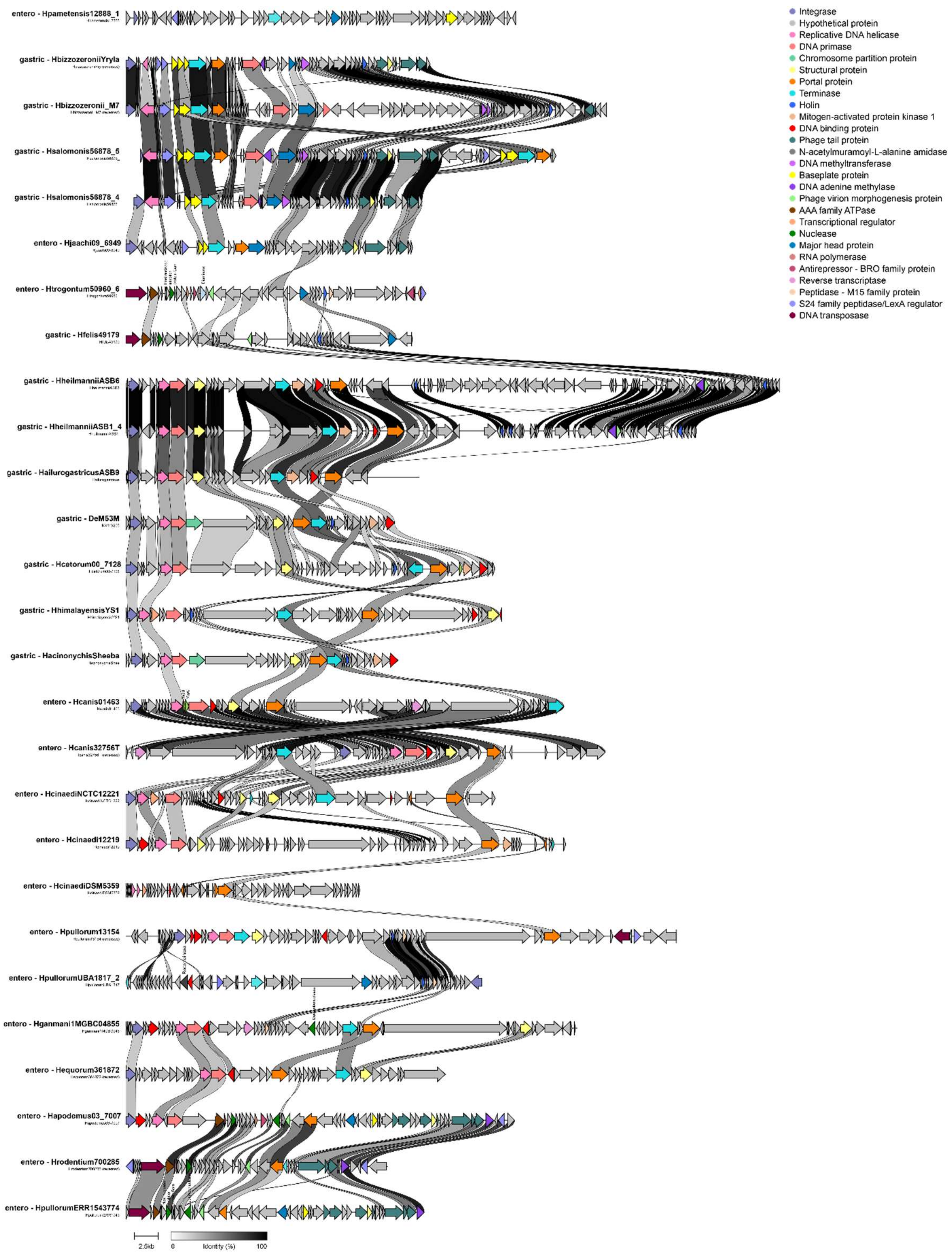

Figure S8. Biosynthetic gene cluster map of representative gastric and enterohepatic species prophages. Figure obtained with clinker and clustermap.js. Arrows represent genes colored by function, and connections represent similarity with minimum alignment sequence identity of 0.3.

Supplementary data

Prophage predicted morphotype

The 124 complete prophages (119 non-pylori *Helicobacter* and 5 *H. pylori* prophages) were analyzed using VirFam to determine their families (Table S2).

Prophages from *H. pylori*, *H. acinonychis*, *H. ailurogastricus*, *H. ganmani*, and *H. himalayensis* were classified as *Podoviridae* type 3. Previously identified *H. pylori* prophages, such as phiHP33, were reclassified from *Siphoviridae* to *Podoviridae*. Some *H. canis*, *H. equorum*, and most *H. cinaedi* prophages could not be classified.

Prophages from *H. felis*, *H. rodentium*, and *H. trogontum* were classified as *Siphoviridae*, while those from *H. apodemus*, *H. jaachi*, *H. pametensis*, and *H. salomonis* were classified as *Myoviridae*. Concerning *H. bizzozeronii*, *H. pullorum*, and *H. heilmannii* each contained prophages from multiple families.

Most gastric prophages are *Podoviridae* (52%), while 83% of enterohepatic prophages were unclassified. Overall, 53% of all prophages were unclassified, with the rest distributed among *Podoviridae* (17%), *Myoviridae* (16%), *Siphoviridae* (8%), and hybrids (5%). The results suggest *Podoviridae* is common in gastric species prophages, but this is not conclusive for enterohepatic species, indicating potential unidentified families.

The TaxMyPhage tool classified all prophages as new species within a new genus, indicating that the results of genomic similarity based on an Average Nucleotide Identity (ANI) was less than 70% compared to the current International Committee on Taxonomy of Viruses (ICTV) classified dsDNA phage genomes.

Prophage defense systems

Among 119 complete *non-pylori Helicobacter* prophages, only a complete defense and a complete anti-defense system were predicted in the set of 119 complete prophages of non-pylori *Helicobacter*. On one hand, the prophage Hpullorum13154 carries a nuclease-helicase (Nhi) [1] defense system, identified in the 5’-end of the prophage. On the other hand, the

prophage Hsp11_5569 encodes a poorly characterized system identified as hia5_hin1523_nma1821_2. This single-protein anti-defense systems is suggested to have anti-restriction-modification activity. Additional 58 putative defense-related genes were identified by HMMer in 38 prophages, with relevance for 15 RM Type_II-associated MTases and 13 PD-Lambda-5_B genes coding for a DNA adenine methylase.

Helicobacter genomes are rich in defense systems, particularly restriction-modification systems, which protect against mobile genetic elements (MGEs) like virulent phages. These systems can also benefit MGEs by aiding their maintenance in bacterial genomes or outcompeting other MGEs and are often carried by prophages [2]. In the arms race between phages and bacteria, phages develop anti-defense systems to evade bacterial defenses [3]. We found only one complete defense and one complete anti-defense system across the entire prophage set. The Nhi defense system, standing for nuclease-helicase immunity, targets and degrades phage genomes at intermediate replication stages [1]. Effective protection against virulent phages was demonstrated independently in *Bacillus* spp. [4] and *Staphylococcus* spp. [1]. Despite rare, it is reported in DefenseFinder database in 0.667% of the analysed *Helicobacter* genomes. Interestingly, the hia5_hin1523_nma1821_2 anti-defense mechanism is suggested to have anti-restriction-modification activity, with hia5, hin1523 and nma1821 genes being previously reported as encoding non-specific DNA adenine methyltransferases in the genome of *H. influenzae* Mu-like prophages [5]. Aligned with this, the carriage of several defense-associated methylases while lacking complementary genes suggest the identified prophages use these genes as part of an evading mechanism to the extremely rich restriction-modification system arsenal typical of *Helicobacter* species [6], rather than an attacking function against other MGEs. In the light of the recent exponential expansion of the defense system topic, it is also not to reject that new, unreported systems and mechanisms can exist in *Helicobacter* genomes, including in their prophages. Nonetheless, some genes associated to abortive infection mechanism or toxin-antitoxin systems, as well as uncharacterized systems, were identified in

the complete prophage genomes, leaving open the possibility that these may play an active role in the multidirectional battle between bacterial hosts and MGEs.

References

1. Bari SMN, Chou-Zheng L, Howell O, Hossain M, Hill CM, Boyle TA, et al. A unique mode of nucleic acid immunity performed by a multifunctional bacterial enzyme. *Cell Host Microbe* 2022; **30**: 570-582.e7.
2. Rocha EPC, Bikard D. Microbial defenses against mobile genetic elements and viruses: Who defends whom from what? *PLoS Biol* 2022; **20**: e3001514.
3. Patel PH, Maxwell KL. Prophages provide a rich source of antiphage defense systems. *Curr Opin Microbiol* 2023; **73**: 102321.
4. Millman A, Melamed S, Leavitt A, Doron S, Bernheim A, Hör J, et al. An expanded arsenal of immune systems that protect bacteria from phages. *Cell Host Microbe* 2022; **30**: 1556-1569.e5.
5. Drozdz M, Piekarowicz A, Bujnicki JM, Radlinska M. Novel non-specific DNA adenine methyltransferases. *Nucleic Acids Res* 2012; **40**: 2119–2130.
6. Vale FF, Mégraud F, Vítor JM. Geographic distribution of methyltransferases of *Helicobacter pylori*: Evidence of human host population isolation and migration. *BMC Microbiol* 2009; **9**.
