# Supplementary material for: Prophage dynamics in gastric and enterohepatic environments: unraveling ecological barriers and adaptive transitions
Source: ISME Commun. 2025 Feb 4;5(1):ycaf017. doi: 10.1093/ismeco/ycaf017 (PMC11840440; doi:10.1093/ismeco/ycaf017)
Supplement: TableS1_ycaf017 [file tables1_ycaf017.pdf]

Genome Name	GenBank Accessions
Candidatus Helicobacter avicola strain 14449	DXIN01000000
Candidatus Helicobacter avistercoris strain CHK158-8274	DXDJ01000000
Helicobacter acinonychis str. Sheeba	AM260522,AM260523
Helicobacter acinonychis strain 212_10	FZLW01000000
Helicobacter acinonychis strain 212_3	FZMD01000000
Helicobacter acinonychis strain 212_4	FZLX01000000
Helicobacter acinonychis strain 212_5	FZLV01000000
Helicobacter acinonychis strain 212_8	FZMC01000000
Helicobacter acinonychis strain 212_9	LT900055
Helicobacter acinonychis strain NCTC12686	UGIA01000000
Helicobacter ailurogastricus strain 157872_3	FZLU01000000
Helicobacter ailurogastricus strain 1578720_6	FZMH01000000
Helicobacter ailurogastricus strain ASB11	CDML01000000
Helicobacter ailurogastricus strain ASB13	CDMH01000000
Helicobacter ailurogastricus strain ASB7	CDMG01000000
Helicobacter ailurogastricus strain ASB9	CDMN01000000
Helicobacter anseris strain 2013ZJHSD2	JXTW01000000
Helicobacter anseris strain MIT 04-9362	NXLX01000000
Helicobacter apodemus MIT-03-7007	JRPC00000000
Helicobacter apodemus strain MGBC162740	CAJURW010000001,CAJURW010000002,CAJURW010000003,CAJURW010000004,CAJURW010000005,CAJURW010000006,CAJURW010000007
Helicobacter apodemus strain SCJK1	CP021886,CP021887
Helicobacter aurati strain 137778_3	FZPM01000000
Helicobacter aurati strain MIT 97-5075	NXLW01000000
Helicobacter baculiformis strain 427351_3	FZMF01000000
Helicobacter bilis ATCC 43879	ACDN00000000
Helicobacter bilis ATCC 49314	JRPI00000000
Helicobacter bilis ATCC 49320	JRPJ00000000
Helicobacter bilis ATCC 51630	JMKW00000000
Helicobacter bilis ATCC 51630	JRPG00000000
Helicobacter bilis Missouri	JRPH00000000
Helicobacter bilis strain 37372_8	FZMS01000000
Helicobacter bilis strain 37372_9	FZMT01000000
Helicobacter bilis strain AAQJH	CP019645
Helicobacter bilis strain ATCC 51630	AZTD01000000
Helicobacter bilis strain CCUG 38895	JAERIZ010000100,JAERIZ010000101,JAERIZ010000102,JAERIZ010000103,JAERIZ010000104,JAERIZ010000105,JAERIZ010000106
Helicobacter bilis strain MGYG-HGUT-01329	CABKOK010000001,CABKOK010000002,CABKOK010000003,CABKOK010000004,CABKOK010000005,CABKOK010000006,CABKOK010000007
Helicobacter bilis WiWa	AQFW00000000
Helicobacter bizzozeronii CCUG 35545	CAGP00000000
Helicobacter bizzozeronii CIII-1	FR871757,FR871758
Helicobacter bizzozeronii strain 10	FZEH01000000
Helicobacter bizzozeronii strain 56877_10	FZMK01000000
Helicobacter bizzozeronii strain ASB22	FZLJ01000000
Helicobacter bizzozeronii strain CIP 105233	JAERIY010000100,JAERIY010000101,JAERIY010000220,JAERIY010000102,JAERIY010000103,JAERIY010000104,JAERIY010000221
Helicobacter bizzozeronii strain Heydar	FZKO01000000
Helicobacter bizzozeronii strain M20	FZLB01000000
Helicobacter bizzozeronii strain R53	FZMY01000000
Helicobacter bizzozeronii strain strain M7	FZLK01000000
Helicobacter bizzozeronii strain Yryla	FZKR01000000
Helicobacter brantae strain MIT 04-9366	NXLV01000000
Helicobacter canadensis MIT 98-5491	ABQS00000000
Helicobacter canadensis MIT 98-5491 (Prj:30719)	ACSF00000000
Helicobacter canadensis strain 123841_4	FZPN01000000
Helicobacter canadensis strain MGYG-HGUT-01348	LR698957
Helicobacter canadensis strain NCTC13221	UGHW01000000
Helicobacter canis NCTC 12740	AZJJ00000000
Helicobacter canis strain CCUG 32756	JAERIX010000001,JAERIX010000003,JAERIX010000004,JAERIX010000002,JAERIX010000008,JAERIX010000012,JAERIX010000013
Helicobacter canis strain CCUG 32756T	VXKE01000000
Helicobacter canis strain MGYG-HGUT-01463	LR698964
Helicobacter canis strain NCTC12410	UGHV01000000
Helicobacter cetorum MIT 00-7128	CP003479.1,CP003480.1
Helicobacter cetorum MIT 99-5656	CP003481.1,CP003482.1
Helicobacter cetorum strain 138563_8	FZMR01000000
Helicobacter cetorum strain 138563_8	FZMW01000000
Helicobacter cetorum strain 138563_8	FZMU01000000
Helicobacter cholecystus strain ATCC 700242	NXLU01000000
Helicobacter cholecystus strain ERZ467480	FZNE01000000
Helicobacter cholecystus strain NCTC13205	LR134518
Helicobacter cinaedi ATCC BAA-847	AP012492.1
Helicobacter cinaedi CCUG 18818	ABQT00000000
Helicobacter cinaedi CCUG 18818 = ATCC BAA-847	BEZM01000000
Helicobacter cinaedi PAGU611	AP012344,AP012345
Helicobacter cinaedi strain 2017D-0197	CP063086
Helicobacter cinaedi strain 213_3	FZMI01000000
Helicobacter cinaedi strain 213_4	FZMJ01000000
Helicobacter cinaedi strain CCUG19503	BGKD01000000
Helicobacter cinaedi strain CCUG19504	BGKE01000000
Helicobacter cinaedi strain D7095	CP063081,CP063082
Helicobacter cinaedi strain DSM 5359	JAERIW010000124,JAERIW010000071,JAERIW010000072,JAERIW010000073,JAERIW010000010,JAERIW010000074,JAERIW010000075

Helicobacter cinaedi strain gHKSHhc_BC_20160917	CP029337
Helicobacter cinaedi strain JCHOHcF01	BHDA01000000
Helicobacter cinaedi strain JCHOHcF02	BHDB01000000
Helicobacter cinaedi strain JCHOHcF03	BHDC01000000
Helicobacter cinaedi strain JCHOHcF04	BHDD01000000
Helicobacter cinaedi strain JCHOHcF05	BHDE01000000
Helicobacter cinaedi strain JCHOHcF06	BHDF01000000
Helicobacter cinaedi strain JCHOHcF07	BHDG01000000
Helicobacter cinaedi strain JCHOHcF08	BHDH01000000
Helicobacter cinaedi strain JCHOHcF09	BHDI01000000
Helicobacter cinaedi strain JCHOHcF10	BHDJ01000000
Helicobacter cinaedi strain JCHOHcS01	BHDK01000000
Helicobacter cinaedi strain JCHOHcS02	BHDL01000000
Helicobacter cinaedi strain JCHOHcS03	BHDM01000000
Helicobacter cinaedi strain JCHOHcS04	BHDN01000000
Helicobacter cinaedi strain JCHOHcS05	BHDO01000000
Helicobacter cinaedi strain JCHOHcS06	BHDP01000000
Helicobacter cinaedi strain JCHOHcS07	BHDQ01000000
Helicobacter cinaedi strain JCHOHcS08	BHDR01000000
Helicobacter cinaedi strain JCHOHcS09	BHDS01000000
Helicobacter cinaedi strain JCHOHcS10	BHDT01000000
Helicobacter cinaedi strain MGYG-HGUT-01432	LR698961
Helicobacter cinaedi strain MRY08-1234	AP017374
Helicobacter cinaedi strain NCTC12219	UGHX01000000
Helicobacter cinaedi strain NCTC12221	UGHZ01000000
Helicobacter cinaedi strain P01D0000	BEYW01000000 AP018676
Helicobacter cinaedi strain P02D0213	BEZO01000000
Helicobacter cinaedi strain P03D0629	BEYX01000000 AP018446
Helicobacter cinaedi strain P04D0736	BEYY01000000
Helicobacter cinaedi strain P05D0741	BEYZ01000000
Helicobacter cinaedi strain P06D0798	BEZA01000000 AP018447
Helicobacter cinaedi strain P07D0876	BEZB01000000
Helicobacter cinaedi strain P08D0905	BEZC01000000
Helicobacter cinaedi strain P09D0927	BEZD01000000
Helicobacter cinaedi strain P10D0937	BEZE01000000
Helicobacter cinaedi strain P11D0946	BEZF01000000
Helicobacter cinaedi strain P11D1015	BEZS01000000
Helicobacter cinaedi strain P12D0946	BEZR01000000
Helicobacter cinaedi strain P13D0979	BEZG01000000
Helicobacter cinaedi strain P14D1067	BEZH01000000
Helicobacter cinaedi strain P15D1072	BEZP01000000
Helicobacter cinaedi strain P16D1106	BEZQ01000000
Helicobacter cinaedi strain P17D1144	BEZI01000000
Helicobacter cinaedi strain P18D1268	BEZJ01000000
Helicobacter cinaedi strain P19D1315	BEZK01000000
Helicobacter cinaedi strain P20D1835	BEZL01000000
Helicobacter cinaedi strain P21D1863	BEZN01000000
Helicobacter cinaedi strain PAGU1382	BGKG01000000
Helicobacter cinaedi strain PAGU617	BGKF01000000
Helicobacter cinaedi strain PAGU628	BGKH01000000
Helicobacter cynogastricus strain 329937_4	FZMQ01000000
Helicobacter equorum strain 361872_4	FZPO01000000
Helicobacter equorum strain MIT 12-6600	NXLT01000000
Helicobacter felis ATCC 49179	FQ670179
Helicobacter felis strain 1602_kol1	FZLC01000000
Helicobacter felis strain 1602_kol2	FZLH01000000
Helicobacter felis strain 1602_kol3	FZLL01000000
Helicobacter felis strain 1602_kol4	FZKQ01000000
Helicobacter felis strain 214_7_1	FZNI01000000
Helicobacter felis strain 2302	FZKU01000000
Helicobacter felis strain cn23e	QXJE01000000
Helicobacter felis strain CS6	FZKM01000000
Helicobacter felis strain CS7	FZKX01000000
Helicobacter felis strain CS8	FZKG01000000
Helicobacter felis strain Dog7	FZLG01000000
Helicobacter felis strain JKM1	FZKL01000000
Helicobacter felis strain JKM2	FZKY01000000
Helicobacter felis strain JKM3	FZKW01000000
Helicobacter felis strain JKM5	FZKZ01000000
Helicobacter felis strain M26	FZKS01000000
Helicobacter felis strain M29	FZLF01000000
Helicobacter felis strain M35KOL	FZKK01000000
Helicobacter felis strain M35KOLBIS	FZLM01000000
Helicobacter felis strain M38	FZKF01000000
Helicobacter felis strain M39	FZKP01000000
Helicobacter felis strain M42	FZLA01000000
Helicobacter fennelliae MRY12-0050	BASD00000000
Helicobacter fennelliae strain DSM 7491	JAERIV010000070,JAERIV010000071,JAERIV010000072,JAERIV010000073,JAERIV010000074,JAERIV010000009,JAERIV010000075
Helicobacter fennelliae strain NCTC11613	UGIB01000000

Helicobacter fennelliae strain NCTC13101	UGID01000000
Helicobacter fennelliae strain NCTC13102	UAWL01000000
Helicobacter ganmani strain MGBC104855	CAJTMP010000001,CAJTMP010000002,CAJTMP010000003,CAJTMP010000004,CAJTMP010000005,CAJTMP010000006,CAJTMP010000007
Helicobacter ganmani strain MIT 99-5101	NXLS01000000
Helicobacter heilmannii ASB1.4	HE984298.2
Helicobacter heilmannii strain 35817_11	FZME01000000
Helicobacter heilmannii strain 35817_15	FZMG01000000
Helicobacter heilmannii strain ASB1	CDMK01000000
Helicobacter heilmannii strain ASB14	CDMI01000000
Helicobacter heilmannii strain ASB2	CDMP01000000
Helicobacter heilmannii strain ASB3	CDMJ01000000
Helicobacter heilmannii strain ASB6	CDMM01000000
Helicobacter heilmannii strain LMG 26292	JAERIU010000100,JAERIU010000101,JAERIU010000102,JAERIU010000103,JAERIU010000104,JAERIU010000105,JAERIU010000106
Helicobacter hepaticus ATCC 51449	AE017125
Helicobacter hepaticus strain MGBC105003	CAJTNB010000001,CAJTNB010000002,CAJTNB010000003,CAJTNB010000004,CAJTNB010000005,CAJTNB010000006,CAJTNB010000007
Helicobacter himalayensis strain YS1	CP014991
Helicobacter japonicus strain MGBC128938	CAJUDB010000001,CAJUDB010000002,CAJUDB010000003,CAJUDB010000004,CAJUDB010000005,CAJUDB010000006,CAJUDB010000007
Helicobacter macacae MIT 99-5501	AZJI00000000
Helicobacter macacae strain MGYG-HGUT-01462	CABKTB010000001,CABKTB010000002,CABKTB010000003,CABKTB010000004
Helicobacter magdeburgensis MIT 96-1001	JRPE00000000
Helicobacter magdeburgensis strain MGBC104683	CAJTLH010000001,CAJTLH010000002,CAJTLH010000003,CAJTLH010000004,CAJTLH010000005,CAJTLH010000006,CAJTLH010000007
Helicobacter marmotae strain 152490_3	FZPP01000000
Helicobacter marmotae strain MIT 98-6070	NXLR01000000
Helicobacter mesocricetorum strain 87012_3	FZPJ01000000
Helicobacter muridarum ST1	JRPD00000000
Helicobacter muridarum strain 216_6	FZMM01000000
Helicobacter muridarum strain 216_7	FZML01000000
Helicobacter muridarum strain 216_8	FZMN01000000
Helicobacter muridarum strain NCTC12714	UGJE01000000
Helicobacter mustelae 12198	FN555004
Helicobacter mustelae strain NCTC12031	UGIC01000000
Helicobacter mustelae strain NCTC12198	LS483446
Helicobacter pametensis ATCC 51478	JADE00000000
Helicobacter pametensis strain 95149_6	FZPI01000000
Helicobacter pametensis strain 95149_7	FZPL01000000
Helicobacter pametensis strain NCTC12888	UYIU01000000
Helicobacter pullorum MIT 98-5489	ABQU00000000
Helicobacter pullorum NCTC 12824 strain CCUG 33837	VZPA01000000
Helicobacter pullorum NCTC 12824 strain CCUG 33837	JAERIT010000025,JAERIT010000010,JAERIT010000026,JAERIT010000011,JAERIT010000012,JAERIT010000013,JAERIT010000014
Helicobacter pullorum strain 2013BJHL	JXTX01000000
Helicobacter pullorum strain 229254/12	JNOA01000000
Helicobacter pullorum strain 229313/12	JNOB01000000
Helicobacter pullorum strain 229334/12	JNOC01000000
Helicobacter pullorum strain 229336/12	JNUR01000000
Helicobacter pullorum strain 35818_8	FZMX01000000
Helicobacter pullorum strain 35818_9	FZMV01000000
Helicobacter pullorum strain Chicken_16_mag_143	CAJFGW010000001,CAJFGW010000002,CAJFGW010000003,CAJFGW010000004,CAJFGW010000005,CAJFGW010000006,CAJFGW010000007
Helicobacter pullorum strain ERR1543774_bin.1_metaWRAP_v1.1_	CAJSFZ010000001,CAJSFZ010000002,CAJSFZ010000003,CAJSFZ010000004,CAJSFZ010000005,CAJSFZ010000006,CAJSFZ010000007
Helicobacter pullorum strain MGYG-HGUT-01312	CABKNZ010000001,CABKNZ010000002,CABKNZ010000003,CABKNZ010000004,CABKNZ010000005,CABKNZ010000006,CABKNZ010000007
Helicobacter pullorum strain NAP10B8	MAOZ00000000
Helicobacter pullorum strain NAP11B31	MAJF00000000
Helicobacter pullorum strain NAP12B32	MAJG00000000
Helicobacter pullorum strain NAP13B35	MANJ00000000
Helicobacter pullorum strain NAP14B36	MANK00000000
Helicobacter pullorum strain NAP1W4	LXWI00000000
Helicobacter pullorum strain NAP2W5	MAPE00000000
Helicobacter pullorum strain NAP3W17	MAPD00000000
Helicobacter pullorum strain NAP5W19	MAPC00000000
Helicobacter pullorum strain NAP6W24	MAPB00000000
Helicobacter pullorum strain NAP8W25	MAPA00000000
Helicobacter pullorum strain NCTC13154	LR134509
Helicobacter pullorum strain NCTC13156	UGJF01000000
Helicobacter pullorum strain SRR5519171-bin.1	CAJKIG010000001,CAJKIG010000002,CAJKIG010000003,CAJKIG010000004,CAJKIG010000005,CAJKIG010000006,CAJKIG010000007
Helicobacter pullorum strain UBA1817	DCEZ00000000
Helicobacter rappini strain 95150_3	FZPK01000000
Helicobacter rodentium ATCC 700285	JHWC00000000
Helicobacter rodentium strain MGBC105438	CAJTNZ010000001,CAJTNZ010000002,CAJTNZ010000003,CAJTNZ010000004,CAJTNZ010000005,CAJTNZ010000006,CAJTNZ010000007
Helicobacter saguini strain 15-1416 (F2) strain 15-1416 (F2) strain 1	CP028938 QBIW00000000
Helicobacter saguini strain 15-1458 (F3) strain 15-1458 (F3) strain 1	CP028937 QBIV00000000
Helicobacter saguini strain 16-048 (F4) strain 16-048 (F4) strain 16-	CP028936 QBIU00000000
Helicobacter saguini strain 97-6194-5 (F0) strain 97-6194-5 (F0) stra	CP028939 QBIX00000000
Helicobacter salomonis strain 56878_3	FZLZ01000000
Helicobacter salomonis strain 56878_4	OANQ01000000
Helicobacter salomonis strain 56878_5	FZLY01000000
Helicobacter salomonis strain 56878_6	FZMA01000000
Helicobacter salomonis strain 56878_7	FZMB01000000
Helicobacter salomonis strain CIP 105607	JAERIS010000053,JAERIS010000054,JAERIS010000010,JAERIS010000055,JAERIS010000011,JAERIS010000012,JAERIS010000013
Helicobacter sanguini MIT 97-6194	JRMP00000000
Helicobacter sp. 10-6591	NHYK01000000

Helicobacter sp. 11-8110	NHYL01000000
Helicobacter sp. 11S02596-1	MLAM00000000
Helicobacter sp. 11S02629-2	MLAN00000000
Helicobacter sp. 11S03491-1	MLAO00000000
Helicobacter sp. 12S02232-10	MLAQ00000000
Helicobacter sp. 12S02634-8	MLAP00000000
Helicobacter sp. 13S00401-1	MLAR00000000
Helicobacter sp. 13S00477-4	MLAS00000000
Helicobacter sp. 13S00482-2	MLAT00000000
Helicobacter sp. 15-1451	NHYN01000000
Helicobacter sp. 16-1353	NHYM01000000
Helicobacter sp. 48519	QXJF01000000
Helicobacter sp. CLO-3	MAMQ00000000
Helicobacter sp. CLO-3	MLQN01000000
Helicobacter sp. 'CLO3_human' strain 106648	FZQU01000000
Helicobacter sp. 'CLO3_human' strain 106650	FZRC01000000
Helicobacter sp. CNRCH 2005/566H	QXJG01000000
Helicobacter sp. Faydin-H64	JAIGYP010000001,JAIGYP010000010,JAIGYP010000011,JAIGYP010000012,JAIGYP010000013,JAIGYP010000014,JAIGYP010000015
Helicobacter sp. Faydin-H70	JAIGYQ010000001,JAIGYQ010000010,JAIGYQ010000011,JAIGYQ010000012,JAIGYQ010000013,JAIGYQ010000014,JAIGYQ010000015
Helicobacter sp. 'house sparrow 1' strain 106649	FZQY01000000
Helicobacter sp. L15	QXQP01000000
Helicobacter sp. L2	QXQT01000000
Helicobacter sp. L4	QXQS01000000
Helicobacter sp. L8	QXQR01000000
Helicobacter sp. L8b	VKGC01000000
Helicobacter sp. L9	QXQQ01000000
Helicobacter sp. MIT 00-7814	NXLL01000000
Helicobacter sp. MIT 01-3238	NXLN01000000
Helicobacter sp. MIT 01-6242	CP016503
Helicobacter sp. MIT 01-6451	JRMQ00000000
Helicobacter sp. MIT 03-1614	JRMS00000000
Helicobacter sp. MIT 03-1616	JROY00000000
Helicobacter sp. MIT 05-5293	JROZ00000000
Helicobacter sp. MIT 05-5294	JRPA00000000
Helicobacter sp. MIT 09-6949	JRPR00000000
Helicobacter sp. MIT 11-5569	JRPB00000000
Helicobacter sp. MIT 14-3879	NXLP01000000
Helicobacter sp. MIT 17-337	NXLQ01000000
Helicobacter sp. MIT 99-10781	NXLM01000000
Helicobacter sp. MIT 99-5507	NXLO01000000
Helicobacter sp. NHP19-0003	AP024814,AP024815,AP024816,AP024817,AP024818
Helicobacter sp. NHP19-0012	AP024819,AP024820,AP024821,AP024822,AP024823,AP024824,AP024825,AP024826
Helicobacter sp. strain Mgla_MAG_31-bin_15	JAAVGY010000001,JAAVGY010000002,JAAVGY010000003,JAAVGY010000004,JAAVGY010000005,JAAVGY010000006,JAAVGY010000007
Helicobacter sp. strain Mgla_MAG_ref_184	JAAVGZ010000030,JAAVGZ010000001,JAAVGZ010000031,JAAVGZ010000076,JAAVGZ010000032,JAAVGZ010000002,JAAVGZ010000033
Helicobacter sp. strain Mgla_MAG_ref_428	JAAVHA010000092,JAAVHA010000036,JAAVHA010000037,JAAVHA010000038,JAAVHA010000002,JAAVHA010000039,JAAVHA010000003
Helicobacter sp. strain RGIG5198	JAFVIJ010000062,JAFVIJ010000063,JAFVIJ010000064,JAFVIJ010000008,JAFVIJ010000009,JAFVIJ010000065,JAFVIJ010000066
Helicobacter sp. strain RGIG5417	JAFVUX010000003,JAFVUX010000004,JAFVUX010000005,JAFVUX010000006,JAFVUX010000007,JAFVUX010000008,JAFVUX010000009
Helicobacter sp. strain UBA8006	DPBQ01000000
Helicobacter sp. strain UBA8859	DPRV01000000
Helicobacter sp. TUL	NESU00000000
Helicobacter sp. UBA3407	DEOX01000000
Helicobacter sp. UBA716	DBLU00000000
Helicobacter suis HS1	ADGY00000000
Helicobacter suis HS1 strain HS1 input strain	FMSU00000000
Helicobacter suis HS1 strain HS1 output strain 1	FMSQ00000000
Helicobacter suis HS1 strain HS1 output strain 2	FMSS00000000
Helicobacter suis HS1 strain HS1 output strain 3	FMST00000000
Helicobacter suis HS1 strain HS1 output strain 4	FMSR00000000
Helicobacter suis HS5	ADHO00000000
Helicobacter suis strain DSM 19735	JAERIR010000100,JAERIR010000101,JAERIR010000102,JAERIR010000103,JAERIR010000104,JAERIR010000105,JAERIR010000106
Helicobacter suis strain HS10	FZKV01000000
Helicobacter suis strain HS2	FZLI01000000
Helicobacter suis strain HS3	FZKT01000000
Helicobacter suis strain HS4	FZKI01000000
Helicobacter suis strain HS5	FZKN01000000
Helicobacter suis strain HS6	FZLD01000000
Helicobacter suis strain HS7	FZKH01000000
Helicobacter suis strain HS8	FZKJ01000000
Helicobacter suis strain HS9	FZLE01000000
Helicobacter suis strain HSMf 331	CABIKG010000001,CABIKG010000002,CABIKG010000003,CABIKG010000004,CABIKG010000005,CABIKG010000006,CABIKG010000007
Helicobacter suis strain HSMf 503b	CABIKI010000001,CABIKI010000002,CABIKI010000003,CABIKI010000004,CABIKI010000005,CABIKI010000006,CABIKI010000007
Helicobacter suis strain HSMf 505/2	CABIKH010000001,CABIKH010000002,CABIKH010000003,CABIKH010000004,CABIKH010000005,CABIKH010000006,CABIKH010000007
Helicobacter suis strain HSMm R02019b	CABIKE010000001,CABIKE010000002,CABIKE010000003,CABIKE010000004,CABIKE010000005,CABIKE010000006,CABIKE010000007
Helicobacter suis strain HSMm R04052c	CABIKF010000001,CABIKF010000002,CABIKF010000003,CABIKF010000004,CABIKF010000005,CABIKF010000006,CABIKF010000007
Helicobacter suis strain HSMm R07055b	CABIKJ010000001,CABIKJ010000002,CABIKJ010000003,CABIKJ010000004,CABIKJ010000005,CABIKJ010000006,CABIKJ010000007
Helicobacter suis strain HSMm R08041b	CABIKK010000001,CABIKK010000002,CABIKK010000003,CABIKK010000004,CABIKK010000005,CABIKK010000006,CABIKK010000007
Helicobacter suis strain NHP19-0020	AP023036,AP023037,AP023038
Helicobacter suis strain NHP19-0033	BLRI01000000
Helicobacter suis strain NHP19-4003	AP023039,AP023040,AP023041

Helicobacter suis strain NHP19-4004	AP023042,AP023043,AP023044,AP023045
Helicobacter suis strain NHP19-4022	AP023046,AP023047,AP023048
Helicobacter suis strain P13/32	CABIUG010000001,CABIUG010000002,CABIUG010000003,CABIUG010000004,CABIUG010000005,CABIUG010000006,CABIUG010000007
Helicobacter suis strain P13/35	CABIUH010000001,CABIUH010000002,CABIUH010000003,CABIUH010000004,CABIUH010000005,CABIUH010000006,CABIUH010000007
Helicobacter suis strain P13/36	CABIUI010000001,CABIUI010000002,CABIUI010000003,CABIUI010000004,CABIUI010000005,CABIUI010000006,CABIUI010000007
Helicobacter suis strain SNTW101	BDAO00000000
Helicobacter suis strain SNTW101c	AP019774,AP019775,AP019776
Helicobacter trogontum ATCC 49310	JRPK00000000
Helicobacter trogontum ATCC 700114	JRPL00000000
Helicobacter trogontum strain 50960_6	FZNG01000000
Helicobacter trogontum strain 50960_7	FZND01000000
Helicobacter trogontum strain 50960_8	FZNF01000000
Helicobacter typhlonius MIT 98-6810	JRPF00000000
Helicobacter typhlonius strain 1	LN907858
Helicobacter typhlonius strain MGBC110213	CAJTQN010000001,CAJTQN010000002,CAJTQN010000003,CAJTQN010000004,CAJTQN010000005,CAJTQN010000006,CAJTQN010000007
Helicobacter typhlonius strain UBA3403	DEPB01000000
Helicobacter typhlonius strain UBA3405	DEOZ01000000
Helicobacter typhlonius strain UBA709	DBMB00000000
Helicobacter valdiviensis strain WBE14	NBIU01000000
Helicobacter winghamensis ATCC BAA-430	ACDO00000000
Helicobacter winghamensis strain 196_13	MBPH01000000
Helicobacter winghamensis strain 2015D-0170	CP063087
Helicobacter winghamensis strain 228_13	MBPI01000000
Helicobacter winghamensis strain 294_13	MBPJ01000000
Helicobacter winghamensis strain 295_13	MBPK01000000
Helicobacter winghamensis strain 296_13	MBPL01000000
Helicobacter winghamensis strain MGYG-HGUT-01330	CABKOI010000001,CABKOI010000002,CABKOI010000003,CABKOI010000004,CABKOI010000005,CABKOI010000006,CABKOI010000007
uncultured Helicobacter sp. strain MGBC103445	CAJTLL010000001,CAJTLL010000002,CAJTLL010000003,CAJTLL010000004,CAJTLL010000005,CAJTLL010000006,CAJTLL010000007
